# Preparation of Zirconium-89 Solutions for Radiopharmaceutical Purposes: Interrelation Between Formulation, Radiochemical Purity, Stability and Biodistribution

**DOI:** 10.3390/molecules24081534

**Published:** 2019-04-18

**Authors:** Anton Larenkov, Victor Bubenschikov, Artur Makichyan, Maria Zhukova, Alina Krasnoperova, Galina Kodina

**Affiliations:** 1 State Research Center—Burnasyan Federal Medical Biophysical Center of Federal Medical Biological Agency, Zhivopisnaya str., bld. 46; 123182 Moscow, Russia; bubenschikov2011@yandex.ru (V.B.); makbezh@gmail.com (A.M.); 9053522@mail.ru (M.Z.); kalina188@mail.ru (A.K.); gkodina@yandex.ru (G.K.); 2 Lomonosov Moscow State University, GSP-1, Leninskie Gory, 119991 Moscow, Russia

**Keywords:** zirconium-89, purification, radiochemical purity, stability, DFO, transchelation, ZR hydroxamate resin, Chelex-100, oxalate, citrate

## Abstract

Zirconium-89 is a promising radionuclide for nuclear medicine. The aim of the present work was to find a suitable method for obtaining zirconium-89 solutions for radiopharmaceutical purposes. For this purpose, the ion exchange behavior of zirconium-89 solutions was studied. Radio-TLC (thin layer chromatography) and biodistribution studies were carried out to understand speciation of zirconium-89 complexes and their role in the development of new radiopharmaceuticals. Three methods of zirconium-89 isolation were studied using ZR (hydroxamate) and Chelex-100 resins. It was found that ZR-resin alone is not enough to obtain stable zirconium-89 formulations. An easy and effective method of reconstitution of [^89^Zr]Zr-oxalate to [^89^Zr]Zr-citrate using Chelex-100 resin was developed. Developed procedures allow obtaining [^89^Zr]Zr-oxalate (in 0.1 M sodium oxalate solution) and [^89^Zr]Zr-citrate (in 0.1–1.0 M sodium citrate solution). These solutions are perfectly suitable and convenient for radiopharmaceutical purposes. Our results prove [^89^Zr]Zr-citrate to be advantageous over [^89^Zr]Zr-oxalate. During evaluation of speciation of zirconium-89 complexes, a new TLC method was developed, since it was proved that there is no comprehensive method for analysis or zirconium-89 preparations. The new method provides valuable insights about the content of “active” ionic form of zirconium-89. The interrelation of the chromatographic behavior of zirconium-89 preparations and their biodistribution was studied.

## 1. Introduction

The development of new radiopharmaceuticals for positron emission tomography (PET) in the aspect of personalized medicine is characterized by incredible progress in recent years [1,2,3]. The number of new compounds highly specific to certain biological processes is constantly increasing [4]. The incredible success of radiopharmaceuticals based on gallium-68 boosted the renaissance of radiopharmaceuticals based on metal radionuclides in general [5,6,7,8,9,10,11]. Labeled peptides with fast accumulation in target organs (comparable to the half-life of gallium-68 (T_½_ = 67.8 min) and similar radionuclides) were particularly effective [6]. However, some highly specific molecules have a sufficiently high molecular weight and, as a result, a long retention in the bloodstream (e.g., monoclonal antibodies [12]). Therefore, researchers focus on radionuclides such as zirconium-89, yttrium-86, gallium-66, scandium-44, and copper-64 [13,14,15,16]. Zirconium-89, with a half-life of 78.42 h, is recently of particular interest. During the decay of zirconium-89, positrons with maximum energy E_β+_ = 0.902 MeV are formed (yield 22.8%). By this positron emission and via electron capture (76.2%), zirconium-89 decays producing yttrium-89m (T_½_ = 15.8 s), which in turn decays to stable yttrium-89 emitting photons (E_γ_ = 909 keV, 99.03%) [17]. The nuclear-physical properties of zirconium-89 make it an extremely promising radionuclide to study processes with a long-term kinetics using PET [18]. Zirconium-89 has several advantages compared to other similar radionuclides. The main advantage is its production feature: to produce zirconium-89 with a cyclotron, an isotopically enriched target is not required, since the starting material of the target is yttrium, which is a monoisotopic element [19]. Another advantage of zirconium-89 is that it has one of the lowest maximum energies of emitted positrons (after fluorine-18, copper-64 and carbon-11), which makes it possible to obtain PET images with high resolution [18,20]. According to the modeling results, which are in good agreement with the published experimental data, ^89^Y(p,n)^89^Zr reaction is considered to be the best for producing zirconium-89, and optimum energy for proton bombardment is 14 MeV [21,22,23,24]. The reaction can be successfully performed using small biomedical cyclotrons (<20 MeV); even 11 MeV cyclotrons are suitable [25,26,27].

There are several thorough reviews on radiopharmaceutical preparations based on zirconium-89 [18,28,29]. One of the earliest studies (it is also probably the first one published) on the distribution of zirconium-89 in human body was performed in 1957. Mealey studied the dynamics of excretion of zirconium-89 from the bloodstream and its distribution between plasma proteins [30]. He reported the possibility of the application of zirconium-89 for the “external localization of human brain tumors”. Zirconium-89 was prepared and administered in the form of [^89^Zr]Zr-citrate (formulated in 1% sodium citrate, pH 6–7). The irradiated target was dissolved in 12 M HCl, passed through an anion exchange resin (Dowex); zirconium-89 was eluted from the resin with 5 M HNO_3_, nitric acid was then evaporated, and zirconium-89 activity was dissolved in in 1% sodium citrate; the solution was filtered to remove colloidal zirconium-89.

Preclinical studies have demonstrated the effectiveness of [^89^Zr]Zr-oxine in studying the distribution of stem cells and leukocytes (including the evaluation the effectiveness of cell therapy) [31], various cancer cells [32], and bone marrow cells after implantation [33]. Park et al. assessed the suitability of [^89^Zr]Zr-oxalate as a PET tracer for the diagnosis of tumor and inflammatory processes, as well as rheumatoid arthritis in an animal model [34]. However, as mentioned above, most zirconium-89 radiopharmaceuticals used and under development today are labeled monoclonal antibodies [35,36]. These radiopharmaceuticals show high suitability in the diagnosis of various oncological diseases using PET. The growing interest in zirconium-89 together with very encouraging data from preclinical and clinical studies can ensure its rapid introduction into routine clinical practice, provided the availability of the radionuclide itself. The long half-life of zirconium-89 makes it possible to effectively implement the concept of its centralized production and delivery to medical institutions, which eliminates the need for a cyclotron-radiochemical complex in the medical institution itself. Thus, the development of effective methods for isolating zirconium-89 and formulating it in solutions that are most convenient for preparing radiopharmaceuticals is an extremely important obstacle on the way of introducing zirconium-89 into medical practice.

For the isolation of zirconium-89 from the irradiated target, cation- [37,38,39] and anion-exchange [23,40,41] chromatography methods, and extraction [38,41,42] were proposed. A typical procedure for processing the target is as follows: The irradiated target is dissolved in concentrated hydrochloric acid (9–12 M HCl). Next, the resulting solution is evaporated and/or diluted to the required concentration of HCl (≥8 M) and passed through an ion exchange column (Dowex 1 × 8, 2 × 8). Desorption of zirconium-89 is carried out with 1–2 M HCl or with combined solutions containing nitric, oxalic or hydrofluoric acid. The yield of zirconium-89 for this purification method ranges from 60% to 98%, depending on the volume and composition of the eluent. The chemical and radionuclide purity of the solutions obtained also varies widely.

In 1965, Baroncelli and Grossi found that hydroxamic acids have high specific affinity for zirconium and form very stable complexes with it [43]. Later, it was shown that hydroxamates have very specific and high affinity for Zr-ions even at high acid concentrations (in contrast to Fe, Al and Y) [44,45]. Thus, these compounds are suitable for a one-step separation of radiozirconium (^88/89^Zr) from the bulk quantities of yttrium and other metal ions present in the target material. Meijs et al. described the first separation of zirconium-89 from yttrium with an in house prepared hydroxamate resin by eluting the no carrier added radiozirconium with 1.0 M oxalic acid [46]. Holland et al. showed that [^89^Zr]Zr-oxalate in the form of 1.0 M oxalic acid solution obtained using hydroxamate resin has high chemical and radionuclide purity, and is suitable for obtaining complexes with deferoxamine with high molar activity (there was no effect of oxalic acid concentration on the yield of the complexation) [47].

Pandya et al. showed that it is possible to obtain zirconium-89 complexes with tetraazamacrocyclic ligands. In that study, [^89^Zr]Zr-DOTA complex showed “extraordinary stability” [48]. It is particularly interesting that the authors demonstrated the effect of the nature of the initial zirconium-89 solution on the complexation efficiency: the use of [^89^Zr]Zr-chloride (solution in 1 M HCl after reconstitution method [47]) when preparing zirconium-89 complexes with various ligands is preferable over the use of [^89^Zr]Zr-oxalate.

It is important to note that hydroxamate resins mentioned in the vast majority of publications are in house prepared. Hence, there may be differences in the experimental data obtained by various scientific groups. ZR-resin (TrisKem International) is probably the only commercially available hydroxamate resin today [49]. According to the data provided by the manufacturer, this resin has a high selectivity for Zr in a wide range of HCl concentrations (0.01–10 M) and in HNO_3_ solutions with concentrations up to 5 M. At the same time, the resin has a low affinity for Y, as well as for Fe(III) in solutions of 1–6 M HCl, and therefore can be effective for the isolation of zirconium-89 from the irradiated target. It is important that zirconium-89 can be eluted from the resin with a solution of oxalic acid with a concentration of 0.05–0.1 M in a small volume (1–1.5 mL). Thus, using this resin, it is possible to decrease the concentration of oxalic acid in the eluent by an order of magnitude with generally accepted methods [46,47]. Unfortunately, today, there are few papers describing the processes of using ZR-resin to isolate zirconium-89. Noteworthy is the work of Graves et al., who carried out the evaluation of the suitability of tributylphosphate(TBP)-impregnated solid-phase resin (TrisKem International) for the isolation of zirconium-89 [50]. As a comparison, a ZR-resin was used. Using TBP-resin, zirconium-89 was obtained in the form [^89^Zr]Zr-chloride (0.1 M HCl). In the case of ZR-resin, zirconium-89 eluted with 1.0 M H_2_C_2_O_4_ (although the manufacturer indicated a concentration of 0.05–0.1 M) and then reconstitution of [^89^Zr]Zr-oxalate to [^89^Zr]Zr-chloride was carried out using the method described in [47]. With TBP-resin zirconium-89 recovery efficiency in 1.0 mL of 0.1 M HCl was 89 ± 3%. At the same time, the content of metallic impurities (Y and Fe) was higher than when using ZR-resin. It is important to note that the preparation of zirconium-89 in dilute hydrochloric acid solutions has a significant drawback. It is known that, in the case of microconcentrations (10^−9^–10^−11^ M ^95^Zr) in solutions of saline, nitric, and perchloric acids, a noticeable hydrolysis of Zr^4+^ ion is observed at concentrations of hydrogen ion below ~2 g-ion/L [51]. The hydrolysis proceeds in steps with the formation of hydroxocations of the formula [Zr(OH)_x_]^4−x^ (x < 3), the monomolecular hydroxide Zr(OH)_4_, and also mixed complex cations and neutral molecules. Obviously, with increasing pH, the depth of the hydrolysis of zirconium-89 in such solutions will increase. The same applies to the reformulation of zirconium-89 in saline. Thus, the preparation of zirconium-89 in solutions of carboxylic acids is preferable in terms of their stability and suppression of zirconium hydrolysis with increasing pH. In addition to ZR-resin, we were also interested in the possibility of using Chelex-100 resin for the formulation of zirconium-89 solutions. The data presented by El-Sweify et al. allow evaluating the suitability of Chelex-100 for reconstitution in carboxylic acids solutions with pH close to neutral [52].

Based on the above, the aim of our work was to find a suitable method for obtaining zirconium-89 solutions in a form convenient for radiopharmaceutical purposes: low acidity, long term stability, suitable for further synthesis of various conjugates, and low or zero (replacement by another suitable intermediate) concentration of oxalic acid.

## 2. Results

### 2.1. Radio-TLC Systems for Analysis of Zirconium-89 Preparations

To date, several radio-TLC systems used for analysis of radiochemical purity of [^89^Zr]Zr-labeled radiopharmaceuticals are mentioned in the literature. These systems are mainly used to analyze [^89^Zr]Zr-labeled monoclonal antibodies to determine unbound zirconium-89 content.

Since iTLC-SG/50 mM DTPA pH 7.0 system is most often mentioned in the literature for analyzing [^89^Zr]Zr-radiopharmaceutical preparations ([^89^Zr]Zr-DFO and [^89^Zr]Zr-DFO-mAb—R*f* = 0; unbound zirconium-89—R*f* = 1) [32,47,53,54,55,56,57,58], we decided to use this system (further referred to as *Method 1*) as a control in all experiments. In addition, according to literature data, zirconium forms more stable complexes with DTPA than those with EDTA (stability constants for [Zr(EDTA)]^0^, [Zr(EDTA)_2_]^4−^, [Zr(DTPA)]^−^ and [ZrH_8_(DTPA)_2_]^2+^ are 27.9 ± 0.1, 54.4 ± 0.2, 35.3 ± 0.3 and 70.3 ± 0.4, correspondingly [59]).

At the same time, it was interesting for us to find a system capable of separating zirconium-89 in the form of an oxalate (or another auxiliary ligand) complex from [^89^Zr]Zr-chloride and its hydrated forms. As a result of a large comparative study, we found a system that is probably the most satisfactory for this purpose. In iTLC-SG/CH_3_OH-H_2_O (1:1), 4% TFA (*v*/*v*) system zirconium-89 in the form of oxalate complex moves with a solvent front (R*f* = 1). At the same time, samples of zirconium-89 in hydrochloric acid solutions of various concentrations (0.1–1.0 M HCl), prepared without using oxalic acid, remain at the start of the chromatogram completely in this system (R*f* = 0) (Table 1; for more detailed information, see Tables S1 and S2 in the Supplementary Materials).

This method (*Method 2*) was used in all further studies in combination with *Method 1*. It is important to note that, when using *Method 2* for analysis, we observed an interesting effect on various [^89^Zr]Zr-oxalate preparations: depending on the pH value and the concentration of oxalic acid in the preparation, either two peaks with R*f* 0.8 and 1.0 or only one of them could be observed. The ratios of the peaks could be different as well. This fact suggests the existence of two dominant forms of zirconium-89 complexes with oxalate anion in these preparations. Based on published data, it can be assumed that probably these forms are [^89^Zr]Zr(oxalate)_4_)^4−^ and [^89^Zr]Zr(oxalate)_3_)^2−^ [60]. A strict correlation between the content of these two forms and the properties of the preparations was not established at current stage of the study. Nevertheless, some regularities in the obtained data were observed:

- The higher was the concentration of oxalic acid in the preparation, the greater the amount of zirconium-89 activity was associated with the peak with R*f* = 1.0 in *Method 2*.
- The higher was the pH value of the preparation (in the range of 1–7), the more zirconium-89 activity was associated with the peak with R*f* = 1.0 in *Method 2*.

There was another interesting effect that we observed in our work when using *Method 1*. With a decrease in the concentration of hydrochloric acid in zirconium-89 solution, an increasing part of the activity remained on the chromatogram origin (see Supplementary Materials, Figure S1). Thus, *Method 1* probably makes it possible to evaluate the relative content of various hydrolyzed forms of zirconium-89 in solutions.

### 2.2. Refinement of Zirconium-89 Solutions with ZR-Resin

In the range of hydrochloric acid concentrations from 2 to 6 M, zirconium-89 was almost completely captured by ZR-resin (97–99%). Losses in washing the column with hydrochloric acid solutions after sorption were extremely low (1%). Table 2 presents the data on the assessment of applicability of various eluents for recovery of zirconium-89 from ZR-resin.

In the case of ZR-resin, recovery of zirconium-89 was achieved only with oxalic acid solutions in contrast to data of Meijs et al. [46]. With solutions of citric, malonic, succinic and acetic acids, zirconium-89 transchelation was negligible. The higher was the concentration of oxalic acid in the eluent, the higher was the yield of zirconium-89. It is worth noting that the concentration of 0.1 M oxalic acid declared by the manufacturer was insufficient for complete extraction of zirconium-89 from the resin.

It is important to note that, when performing both experiments on zirconium-89 isolation and model experiments, the eluates after the column filled with ZR-resin were pale yellow (especially solutions of hydrochloric acid). Moreover, significant changes were seen in the UV spectra of model solutions of eluents (both solutions of hydrochloric and oxalic acids, and water) before and after contact with ZR-resin. The higher was the concentration of oxalic acid in the eluent, the lower was the minimum volume of the zirconium-89 eluate (see Supplementary Materials, Figure S2).

There was an interesting correlation in the chromatographic behavior of [^89^Zr]Zr-oxalate samples obtained using ZR-resin with different concentrations of oxalic acid in the eluent: the higher was the concentration of oxalic acid in the eluent, the higher was the amount of zirconium-89 activity moving with the front of the solvent during the analysis according to *Method 1*. This fact probably indicates that, as the concentration of oxalate anions in the sample increases, less and less zirconium-89 undergoes hydrolysis. Another interesting fact is that, as mentioned above, the higher was the concentration of oxalic acid in the sample, the greater part of zirconium activity was associated with a peak having R*f* 1.0 in *Method 2* (for more detailed information, see Table S3 in the Supplementary Materials).

The most interesting is the fact that samples of [^89^Zr]Zr-oxalate preparations, obtained using ZR-resin, show chromatographic behavior that differs from that of samples of [^89^Zr]Zr-oxalate preparations obtained using the same oxalic acid concentration, but prepared from the origin solution [^89^Zr]Zr-cloride (Dowex) (see Supplementary Materials, Table S4). When analyzing [^89^Zr]Zr-oxalate samples, obtained using ZR-resin, using *Method 1*, the higher was the pH, the greater was the amount of the activity remains at the origin of the chromatogram. This was probably due to an increase of zirconium-89 fraction that underwent hydrolysis. It is remarkable that [^89^Zr]Zr-oxalate samples with the same concentration of the oxalic acid, but prepared from the original [^89^Zr]Zr-chloride solution, also followed this trend, but it was much less intense. It is not clear what this difference is connected with the presence of an organic impurities that get into the preparations when using ZR-resin or the difference in the set of zirconium-89 oxalate complexes in various methods of preparation. It may also be influenced by differences in ionic strength and in particular the differences in concentrations of chlorine ions in the formulations. Reference samples (without ZR-resin) were prepared from the origin [^89^Zr]ZrCl_4_ solution in 5 M HCl (the solution was diluted with water to a concentration of hydrochloric acid of 1 M; an aliquot of a concentrated aqueous solution of oxalic acid of necessary concentration was added to achieve 0.1 M oxalate solution; and then, pH was adjusted with NaOH solution). Thus, the concentration of chloride ions in the reference samples was significantly higher than in the samples after the ZR-resin (other parameters being equal).

However, when adding concentrated aqueous solution of sodium chloride to [^89^Zr]Zr-oxalate samples, obtained using ZR-resin, we did not observe any significant changes in the chromatographic behavior. It is worth noting that, in our experiments, we reached the concentration of sodium chloride of 0.4 M in the final preparation. Whether the increase of sodium chloride concentration up to 1.0 M would produce an observed effect on the behavior of the preparation is a matter of further research.

### 2.3. Refinement of Zirconium-89 Solutions with Chelex-100 Resin

It has been shown that zirconium can be sorbed on a Chelex-100 resin from an oxalic acid solution at low pH values [52]. With pH 0–1, the distribution coefficient of zirconium on Chelex-100 was about 500 mL/g for 0.1 M oxalic acid (about 10 mL/g for citric acid). With pH increasing, the value of the distribution coefficient dropped significantly and reached a value close to 1 mL/g if pH > 2 for citric acid and at pH ≥ 4 for oxalic acid. Based on the data presented, we assumed that zirconium-89 can be effectively sorbed on Chelex-100 resin from oxalic acid solutions (obtained after a hydroxamate resin), and then effectively desorbed with solutions of sodium oxalate or sodium citrate. Experimental data confirmed our assumption and showed that, when [^89^Zr]Zr-oxalate solutions in 0.1 M oxalic acid (obtained after ZR-resin) were passed through a column filled with Chelex-100 resin, 98 ± 1% of the activity remained on the column. Zirconium-89 could then be effectively eluted from the column with 0.1 M sodium oxalate solution (~97%) in 500–600 μL. Moreover, the activity loss at this stage could be reduced to a negligibly small portion (<0.5%) when using 0.1 M oxalic acid to wash the column after zirconium-89 sorption. However, when using 0.1 M HCl for the same purpose, the loss during washing was higher—2–3%.

**Figure 1:**
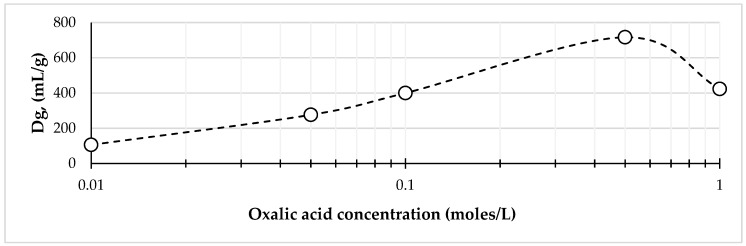
Dependence of zirconium-89 distribution coefficients (*D*g) on oxalic acid concentration for Chelex-100 resin.

Experiments on zirconium-89 sorption on Chelex-100 resin in static conditions were carried out at time points of 5, 10, 20, 30, and 60 min. The results show that after 20 min there were no further changes in the distribution of zirconium-89 between the phases. Therefore, the time point of 30 min was chosen for distribution coefficients determination. Experiments on the effect of oxalic acid concentration on the distribution coefficients (*Dg*) of zirconium-89 on the Chelex-100 showed that this dependency had a maximum, and the highest *Dg* ~ 700 was reached at 0.5 M oxalic acid concentration (Figure 1).

**Figure 2:**
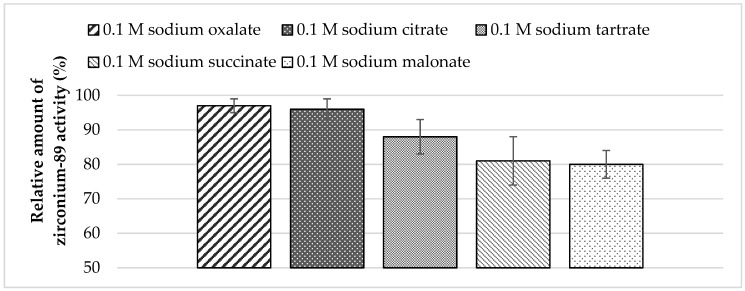
Dependence of zirconium-89 elution efficiency using different sodium carboxylates solutions as eluents with Chelex-100 resin (load from 0.5 M oxalic acid medium: losses at the sorption step—0.7%; losses at the washing step (0.1 M oxalic acid)—0.3%).

To recover zirconium-89 from Chelex-100, resin solutions of sodium salts of various carboxylic acids were assessed. Experimental data show that the highest yield in activity could be achieved using sodium oxalate and citrate solutions (Figure 2).

When using 0.1 M Na_3_Citr as an eluent, 94 ± 2% of sorbed zirconium-89 activity could be obtained in 350–400 μL (see Supplementary Materials, Figure S3).

**Figure 3:**
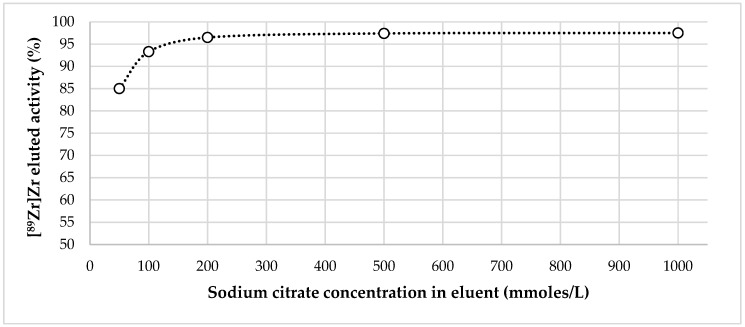
Dependence of zirconium-89 elution efficiency from Chelex-100 on sodium citrate concentration in eluent.

Figure 3 demonstrates the dependency of zirconium-89 recovery from Chelex-100 on the concentration of sodium citrate in the eluent.

As it can be seen from the experimental data, when the concentration of sodium citrate in the eluent was 0.05 M elution, efficiency was about 85%. Starting with 0.2 M, it reached its maximum (97 ± 2%) and then plateaued. Bearing in mind the data of El-Sweify et al. [52], it is highly probable that this dependence is actually associated not so much with a change in the actual concentration of sodium citrate, as with a change in the pH value of eluents after being passed through the column. For example, pH of 1.0 M sodium citrate solution was 9.2 before entering the column and 6.8 after leaving the column, and pH of 0.1 M sodium citrate solution was 8.9 before entering the column and 5.1 after leaving the column (given that after [^89^Zr]Zr-oxalate in 0.1 M oxalic acid (obtained after ZR-resin) the column was rinsed with 0.1 M HCl).

In terms of the application of Chelex-100 resin for converting zirconium-89 to the non-oxalate form, washing the resin after passing oxalic acid solutions is important. Comparison of various washing solutions showed that the best results could be obtained using 0.1 M HCl–0.1 M H_3_Citr (1:1) for washing the column. Losses of the activity at the washing stage in this case were negligible (≤0.5%) (for more detailed information, see Figure S4 in the Supplementary Materials).

It is important to note that, when [^89^Zr]Zr-citrate preparations in 0.1–1.0 M citric acid obtained after Chelex-100 resin were analyzed using TLC *Methods 1* and *2*, two features were observed:

(1) When using *Method 1*, the peak that moved with the solvent front was often characterized by the significant tailing, which complicated the interpretation of the chromatogram (this method is probably not the best one for preparations such as these).

(2) When using *Method 2*, the chromatographic pattern was sharp enough and no “doubling” of the peak was observed at the solvent front (which took place when evaluating [^89^Zr]Zr-oxalate preparations) (see Supplementary Materials, Table S5).

### 2.4. Comparison of Zirconium-89 Preparations Obtained with Different Methods

Based on the data presented in this paper, we propose three methods for preparation of zirconium-89 solutions (for more detailed information, see Figure S5 in the Supplementary Materials):

- using ZR-resin only (0.1–0.115 M oxalate anion concentration; 0.5 M oxalic acid is necessary if the combined method is further implemented);
- combination of ZR-resin and Chelex-100 (0.1–0.116 M oxalate-anion concentration); and
- combination of ZR-resin and Chelex-100 (0.1–1.0 M citrate-anion concentration).

For ease of further interpretation of the results, the term “radiochemical impurities” of zirconium-89 solutions for radiopharmaceutical purposes should be introduced, by analogy with other solutions of radionuclides for labeling (e.g., “Gallium (^68^Ga) chloride solution for radiolabeling” or “Yttrium (^90^Y) chloride solution for radiolabeling”; EDQM, Ph. Eur.). “Radiochemical impurities” assumed to be the sum of all zirconium-89 forms that are “inactive”, i.e., cannot participate in labeling reaction (colloidal form).

Bearing in mind that radiochemical purity is the proportion of the total radioactivity in the sample, which is present as the desired radiolabeled species, in the case of zirconium-89 in radiopharmaceutical preparations, radiochemical purity is the proportion of zirconium-89 presented in “active” form. Regardless of method used to prepare a sample of zirconium-89, for a number of the preparations, there is one important stability feature to be noted. If according to *Method 1* RCP ≤ 100% and according to *Method 2* RCP = 100%, then over time RCP (*Method 2*) remains equal to 100% while RCP (*Method 1*) drops to 0%. As soon this situation occurs (RCP (*Method 1*) = 0%, RCP (*Method 2*) = 100%), the zirconium-89 activity presented in the solution begins to drop, being settled on the surface of the vial (in our case, Eppendorf polypropylene tubes). In this case, further changes in the chromatographic picture according to both methods were not observed. In 24 h, the amount of zirconium-89 remaining on the surface of the vial (in the case of quantitative transfer of the entire volume of the preparation) is in the 20–40% range.

This settled on the surface activity of zirconium-89 can be easily returned to the solution by adding 0.1 M HCl. As a rule, when formulating preparations of zirconium-89 in an oxalate solution, if the RCP value according to *Method 1* was in the range of 95–99%, then its drop in time to 0% was extremely slow (more than 14 days). If this RCP value was in the range of 90–95%, then the drop took about 9–10 days. With RCP less than 90%, the drop took 2–7 days. Thus, for the preparations obtained by each of the proposed methods, the maximum pH value was the one allowing obtaining preparations with RCP (*Method 1*) ≥ 90%, RCP (*Method 2*) ≥ 99%. When studying the stability of the preparations, we decided that a preparation could not be stored beyond the moment when the following values were achieved: RCP (*Method 1*) = 0%, RCP (*Method 2*) = 100% (and in any case if RCP (*Method 2*) ≤ 99%). For an example of changing the chromatographic behavior of [^89^Zr]Zr-oxalate preparations, obtained using various methods, during their storage, see Supplementary Materials, Table S6.

It is noteworthy that, unlike [^89^Zr]Zr-oxalate samples obtained using ZR-resin, samples obtained using Chelex-100 resin exhibited chromatographic behavior similar to that of [^89^Zr]Zr-oxalate preparations obtained from the original zirconium-89 solution in HCl by adding oxalic acid with further neutralization (see Supplementary Materials, Table S4). The main characteristics of the proposed processes are presented in Table 3.

Over time, only for a zirconium-89 obtained in the form of 0.1 M sodium citrate solution (pH 6.5), the formation of an impurity (colloidal zirconium-89 hydroxide) was observed using *Method 2*. For all other methods of preparation RCP (*Method 2*) ~ 100%, no changes in time were observed. It is noteworthy that, with the same concentration of oxalate anions in the solution, the preparations obtained with the combination of ZR-resin + Chelex-100 made it possible to achieve higher pH values (6.5 ± 0.5) in the final solution, compared with the method of using only ZR-resin (pH 5.0 ± 0.5). In addition, these solutions were stable for a much longer time. This effect could be related to the presence of organic impurities. Another possible explanation is the difference between forms of zirconium-oxalate complexes presented in the preparations obtained using ZR and Chelex-100 resins. This is the subject of further research. At this stage of research, the method using ZR-resin only was left solely for comparison.

**Figure 4:**
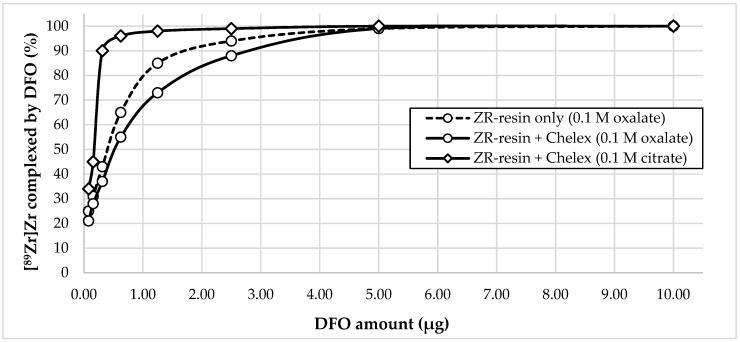
The dependence of [^89^Zr]Zr-DFO complex formation yield on the amount of chelator for various formulations of zirconium-89.

To assess the suitability of zirconium-89 preparations obtained using the proposed methods for the synthesis of radiopharmaceuticals, we carried out experiments to evaluate the efficiency of complexation of zirconium-89 in these preparations with DFO (estimated using the DFO titration method). The pH of the reaction mixture for each preparation was 5.5 (i to compare the combined methods (ZR-resin + Chelex-100) with ZR-resin only method). The incubation time was 15 min. The volume activity of zirconium-89 in all samples was 4.0 ± 0.5 MBq/mL. The volume of the reaction mixture was 1 mL. The results of the experiment are presented in Figure 4.

Experimental data show that, for all three zirconium-89 preparations with 5 μg DFO, a quantitative yield of the complexation reaction was observed (99–100%). However, with a decrease in the chelator concentration, the difference in the efficiency of complexation for the three formulations of zirconium-89 became apparent. An amount of 2.5 μg allowed achieving a complexation yield of the zirconium-89 preparation using ZR-resin only procedure (0.1 M oxalic acid) of 94%; for ZR-resin + Chelex-100 procedure (0.1 M sodium oxalate), the complexation yield was 88%; and, for ZR-resin + Chelex-100 procedure (0.1 M sodium citrate), the complexation yield was 99%. In general, zirconium-89 preparations, formulated in 0.1 M sodium citrate, showed greater efficiency of complexation than oxalate preparations. It is noteworthy that, in the preparations of zirconium-89 in oxalate solution, the preparations obtained by the combined ZR-resin + Chelex-100 method (0.1 M sodium oxalate) showed a lower efficiency of complexation than the obtained ZR-resin only procedure (0.115 M oxalic acid). To a certain extent, this is consistent with the resulting difference in the stability of these solutions. One possible explanation may be that the preparations obtained by the combined ZR-resin + Chelex-100 procedure (0.1 M sodium oxalate) and ZR-resin only procedure (0.1 M oxalic acid), even under the same conditions of oxalate concentration and pH values, contain different mixtures of complexes of zirconium-89 and oxalate anion with different stabilities.

According to our data, to achieve a quantitative yield of the [^89^Zr]Zr-DFO formation reaction, a much larger amount of chelator was needed than, for example, presented by Holland et al. [47]. Probably, this difference was due to the non-optimal pH value chosen in our case (pH 5.5, while, according to the literature data, pH ~ 7 is optimal for the formation of [^89^Zr]Zr-DFO complex). Nevertheless, very encouraging results were obtained using zirconium-89 formulated in sodium citrate solutions (0.1–1.0 M). pH 5.5 was used to observe the conditions under which the chromatographic behavior of the applied zirconium-89 solutions is the same.

It is important to note that *Method 2* was not suitable for analyzing reaction mixtures in the case of labeling reactions, when one or another chelating agent (for example, DFO) was added to the zirconium-89 solution (see Supplementary Materials, Table S7). In this system, there was no difference in the chromatographic behavior of both the initial solution of zirconium-89 (blank solution) and the reaction mixture with the addition of DFO (no matter how well the complexation proceeded according to the results of the analysis using *Method 1*). This may be due either to the fact that the [^89^Zr]Zr-DFO complex in it had the same mobility as [^89^Zr]Zr-oxalate (Rf = 1.0), or [^89^Zr]Zr-DFO complex quickly and completely degraded when contacting the eluent (which is less likely).

Another important aspect is the fact that, if [^89^Zr]Zr-oxalate was used for complexation, the data obtained by *Method 1* and by iTLC-SA/50 mM EDTA (pH 5) method practically coincide (since *Method 2* does not allow evaluating the efficiency of complexation reaction, we decided to supplement the analysis of samples containing DFO with one more method). However, in the case of using [^89^Zr]Zr-citrate solutions, the iTLC-SA/50 mM EDTA system (pH 5) also turned out to be ineffective. Of course, to some extent, yields of [^89^Zr]Zr-DFO formation could be evaluated by the shape of the peak at the start of the chromatogram (which became much narrower), but this was not a sufficient analytical signal. Thus, the question of selecting adequate systems for analyzing various radiopharmaceutical preparations based on zirconium-89 remains a sticking point.

Since today there are publications describing the possibility of synthesizing zirconium-89 complexes with macrocyclic ligands (DOTA, etc.) exhibiting high stability [48,50], and such reactions require heating, we studied the stability of the preparations obtained by using zirconium-89 when heated. From the results, zirconium-89 preparations in 0.1 M sodium citrate solution (pH 6) did not withstand heating, in contrast to 1.0 M sodium citrate solutions and 0.1 M sodium oxalate solutions with the same pH values. After 30 min of incubation at 95 °C, when analyzed by both TLC methods, a large fraction of zirconium-89 activity remained at the start of the chromatogram (~45% in *Method 1*; ~40% in *Method 2*). This fact is likely evidence of the hydrolysis of zirconium-89 occurring under the given conditions (for more detailed information, see Table S8 in the Supplementary Materials). In the case of the use of these samples for further labeling of DFO, this fact is not a significant drawback, since the complexation reaction proceeded at room temperature. In general, heating was not possible at all in direct labeling of monoclonal antibodies with zirconium-89. However, in relation to obtaining zirconium-89 complexes with macrocyclic chelators (DOTA, etc.), the procedures for the preparation of zirconium-89 solutions proposed in this study were unsuitable. In this case, it was necessary to use 1.0 M sodium citrate solution. For samples of zirconium-89 formulated in 0.1 M sodium citrate solution, temperature sterilization was also unacceptable.

### 2.5. Relation between Chromatographic Behavior of [^89^Zr]Zr-Oxalate Preparations and Their Biodistribution

In the course of this work, as noted above, we repeatedly noted a significant difference in the chromatographic behavior of zirconium-89 preparations in various chromatographic systems. The difference between results obtained in *Method 1* and *Method 2* is of particular interest.

Quite often, especially in the pH range of 5–8 and with low oxalate anion concentrations (≤0.5 M), when analyzing preparations using *Method 1*, the greater activity peak (up to 100%) was detected at the start of the chromatogram. This should count in favor of the fact that zirconium-89 in the preparation underwent hydrolysis and was not capable of further complexation. At the same time, when analyzing the same samples according to *Method 2*, all the activity applied to the chromatographic strip moved with the solvent front. The first thing assumed was the idea that *Method 1* showed the presence of colloidal zirconium-89 and, in *Method 2*, the dissolution of these hydrated zirconium-89 forms occurred due to the presence of acid (4% TFA (*v*/*v*). This dissolved fraction of zirconium-89 was then transformed into the ionic form. Another assumption was that *Method 1*, for all its popularity in the scientific literature, did not reflect the nature of zirconium-89 forms in samples adequately, in contrast to *Method 2*. It is known that if a metal radionuclide in a preparation (in radiopharmaceutical) undergoes hydrolysis to form a colloidal form, there will be two observations to be made during further experiments. Firstly, this form will obviously remain at the start of the radio-chromatogram when analyzed by thin-layer chromatography. Secondly, when administered to animals, there will be an accumulation of the activity in the liver and, less frequently, in the lungs as a result of the seizure of colloids by cells of the reticular-endothelial system.

To establish the correlation between the chromatographic behavior of zirconium-89 preparations and their biodistribution, a series of biological experiments were conducted. Various preparations were introduced to laboratory animals. The result of these experiments allowed assessing the adequacy of the chromatographic systems used.

Table 4 presents the results of the biological distribution of three representative preparations of [^89^Zr]Zr-oxalate.

The results of radio-chromatography show that, in Sample 1, according to both methods, all zirconium-89 was in the “active” ionic form and its RCP was about 100%. In Sample 2, according to the results of *Method 1*, all zirconium was in “inactive” form (highly hydrated, RCP ~ 0%), while *Method 2* still indicated RCP of 100% in the preparation. The results of direct radiometry of the mice organs 1 and 24 h after intravenous administration show that the difference in the biological behavior of Samples 1 and 2 was absent (within error margins). No increase in Sample 2 accumulation in the liver was observed compared to Sample 1. In Sample 3, *Method 1* chromatography data demonstrate that its RCP was 0%. According to *Method 2*, ~60% of Sample 3 activity stayed at the origin of chromatogram. This part of zirconium-89 activity could be associated with the presence of zirconium-89 in colloidal form. The direct radiometry data show a significant difference in the biodistribution of this sample compared to Samples 1 and 2. As expected, the accumulation of the preparation occurred mainly in the liver. The accumulation in other organs and tissues for this preparation was significantly lower than that of Samples 1 and 2. This fact suggests that *Method 2* gave a real indication of the presence or absence of zirconium in the preparation in the “active” ionic form. The suitability of *Method 1* in this case was extremely doubtful.

**Figure 5:**
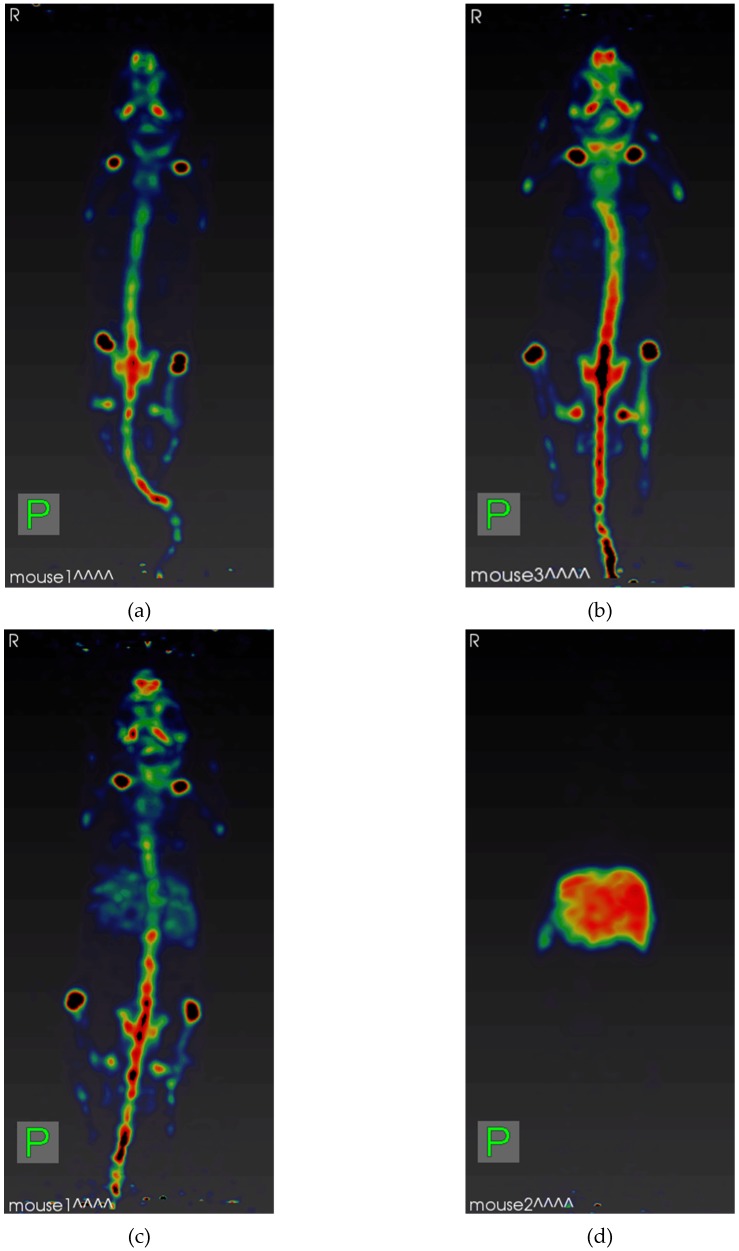
μPET maximum intensity projections of mice after 24 h p.i. of [^89^Zr]Zr-oxalate samples listed at Table 5: (**a**) Sample 1; (**b**) Sample 2; (**c**) Sample 3; and (**d**) Sample 4.

Another series of experiments (Table 5 and Figure 5) gave a more visual representation in the difference in the biodistribution of zirconium-89 samples, since laboratory animals were studied using μPET. Table 5 presents the chromatographic data of the most representative samples.

As in the previous series of experiments, Sample 1 had an RCP of 100% according to both methods. Sample 2 according to the results of *Method 1* had an RCP ~ 0%, whereas, according to *Method 2*, its RCP was 100%. The results of µPET 24 h after administration (Figure 5a,b) of these preparations indicate that there was no difference in the biodistribution of these samples: only the skeleton was visualized on tomograms. The accumulation was increased in the areas of active physiological bone tissue metabolism (bone epiphyses) in both cases. Samples 3 and 4, according to *Method 1*, had zero RCP. At the same time, the results of *Method 2* indicate that in Sample 3 about 10% of zirconium-89 might be presented in hydrolyzed form, and in Sample 4, almost all of it was present in hydrolyzed form.

The results of µPET 24 h after Sample 3 administration (Figure 5c) indicate that most of the accumulated activity was associated with the bone tissue of the animal, but the liver is also clearly visualized on the tomogram. On a tomogram obtained 24 h after Sample 4 administration (Figure 5d), only the liver can be seen.

At this stage, we can confidently say that determining the content of unbound zirconium-89 in radiopharmaceutical preparations ([^89^Zr]Zr-labeled antibodies) using *Method 1* did not provide an adequate estimation of unbound zirconium-89 forms. *Method 2* gave more complete and adequate estimation of the content of zirconium-89 forms in a preparation. Using this method, the presence of “inactive” hydrolyzed forms of zirconium-89 could be clearly evaluated.

### 2.6. Reconstitution of Zirconium-89 From the Oxalate Form into the Chloride Form Using an Anion-Exchange Cartridge

Pandya et al. showed that, during the synthesis of zirconium-89 complexes with DOTA, DOTAM and DOTP macrocyclic ligands, the yield of complexation is much lower when [^89^Zr]Zr-oxalate solutions are used in the reaction compared to using [^89^Zr]Zr-chloride ([^89^Zr]ZrCl_4_) solutions [48]. Thus, when using [^89^Zr]Zr-oxalate for 120 min at a temperature of 99 °C and pH 7.0, the yield of the complexation reaction was 65 ± 9.6%. The authors used the scheme of the reconstitution of [^89^Zr]Zr-chloride into [^89^Zr]Zr-oxalate using the Sep-Pak QMA anion-exchange cartridge, as proposed in [47]. After using [^89^Zr]ZrCl_4_ solution obtained by this method in the reaction, the yield of complexation with DOTA was quantitative (~100%, 90 °C, 40 min, pH 7.0).

In our study, we found an interesting effect. We repeated the reconstitution process described, but, instead of the Sep-Pak QMA cartridge, we used the Chromafix 30PS-HCO_3_ cartridge (see Supplementary Materials, Figure S6).

Zirconium-89 was almost quantitatively (≥99.0%) sorbed on Chromafix 30PS-HCO_3_ cartridge from 0.1–1.0 M solution of oxalic acid (after ZR-resin). After washing with water, about 97 ± 2% of the initial activity of the oxalate solution was desorbed from the cartridge in 500 μL of 1.0 M HCl. It is interesting that, when analyzing the zirconium-89 solutions obtained in this way (*Method 2*), all the activity of the sample moved along with the solvent front. At the same time, when choosing this system, we made a comparison, and not a single sample of zirconium-89, obtained in hydrochloric acid solution without the use of oxalic acid, showed a similar chromatographic behavior. Even more interesting is the fact that the solutions obtained by this reconstitution method showed greater stability when neutralized to a pH range of 5–7 than oxalate-free solutions of zirconium-89 with the same initial concentration of HCl (for more detailed information, see Tables S2, S9 and S10 in the Supplementary Materials).

In the work of Holland et al., who proposed a reconstitution method, it is indicated that it allows removing > 99.8% of the initial amount of oxalic acid [47]. However, some amount (albeit extremely small) of oxalic acid still gets into the final solution. Our data indicate that even these extremely small amounts of oxalate anion can drastically change the chromatographic behavior of zirconium-89 preparations. Probably, zirconium-89 in this preparations was still in the form of a complex with oxalic acid, but that complex might have a different composition (possibly [^89^Zr]Zr(oxalate)^2+^ and/or [^89^Zr]Zr(oxalate)_2_^0^*_aq._*). Thus, it is likely that the difference in the yield of complexation of zirconium-89 with macrocyclic ligands, shown in [48], may be due not only to the transformation of zirconium-89 from one to another chemical form, but also due to the influence of the concentration of oxalate anion itself on the reactivity these chelators. In addition, the observed difference in the measured average apparent molar activity of obtained [^89^Zr]Zr-DOTA complexes for TBP-separated [^89^Zr]Zr-chloride (oxalate-free) and QMA-reconstituted [^89^Zr]Zr-chloride [50] is probably related to the presence of residual oxalic acid and the presence of zirconium-89 in the form of oxalate complexes after the reconstitution (and, therefore, with the degree of hydrolysis of zirconium-89).

Considering the difference in the chromatographic behavior of zirconium-89 preparations obtained in hydrochloric acid by various methods (oxalate and reconstitution), it is logical to assume that, in the preparations of zirconium-89 formulated with an anion-exchange cartridge, zirconium-89 was still present in the form of oxalate complexes. However, those complexes might have a different composition, and, as a result, a different stability. Considering the above, further studies on determining (specifying) the constants of the stability of zirconium complexes with DFO, DOTA (and analogs) are very important, and so is studying the effect of a buffering agent in the reaction mixture on the distribution of zirconium-89 forms, as well as the influence of the nature of the counterion in the case of reconstitution of zirconium-89 solutions using anion-exchange cartridges.

## 3. Discussion

The implementation of radiopharmaceuticals based on zirconium-89 (especially labeled monoclonal antibodies) into clinical practice will allow us to bring the effectiveness of radionuclide diagnostics of various diseases to a new level. The growing interest in this radionuclide, the development of new chelating agents for it, and preclinical and clinical studies of various [^89^Zr]Zr-based radiopharmaceuticals suggest its high potential for the needs of nuclear medicine. Therefore, the question of obtaining solutions of zirconium-89 in a suitable and convenient form for radiopharmaceutical purposes is extremely important.

Meijs et al. described the first separation of zirconium-89 from yttrium with an in house prepared hydroxamate resin by eluting the n.c.a. radiozirconium with 1.0 M oxalic acid [46]. The authors studied different eluents (oxalic acid, acetate and citrate solutions) for ^88/89^Zr removal from the resin, and found oxalic acid to have the best elution efficiency. Increasing the oxalic acid concentration from 0.01 to 1.0 M raised the percentage of zirconium-89 eluted from the column from ~20% to 98% (with oxalic acid concentration of 0.5 M the elution of zirconium-89 was nearly quantitative (95%)). The ability of citrate to remove radiozirconium from “hydroxamate material” was found to be not only concentration- but also pH-dependent: at pH 4.0, transchelation occurred (80% at 1 M), but, at lower pH values, the transchelating capacity was less. It also decreases smoothly with a decrease of citrate concentration in the eluent. Holland et al. showed that [^89^Zr]Zr-oxalate in the form of 1.0 M oxalic acid solutions obtained using hydroxamate resin have high chemical and radionuclide purity, and are suitable for obtaining complexes with deferoxamine with high molar activity (no effect of oxalic acid concentration on the yield of the complexation) [47]. The yield of complexation was close to quantitative at a concentration of deferoxamine higher than 0.03 μg (0.04–0.07 μg). Elution of zirconium-89 from hydroxamate resin was achieved with 99.5% recovery of the radioactivity by transchelation with 1.0 M oxalic acid. Attempts to elute the activity with lower than < 0.5 M had no effect. Only partial recovery of zirconium-89 activity (13–34%) was achieved by using oxalic acid concentrations of 0.5–0.75 M. Later, O’Hara et al. showed that, at 0.8 M H_2_C_2_O_4_, K*d* for zirconium-89 was < 2 mL/g and 0.8 M H_2_C_2_O_4_ eluent solution concentration is adequate [61].

ZR-resin (TrisKem International) is probably the only commercially available hydroxamate resin today [49]. It is important that according to the data provided by the manufacturer zirconium-89 can be eluted from the resin with a solution of oxalic acid with a concentration of 0.05–0.1 M (less than in generally accepted methods [46,47]) in a small volume (1–1.5 mL). It should be noted that according to the literature the solution with a sodium oxalate concentration of 1.55% (0.116 M) is isotonic [62,63]. When using a solution of this composition, there is no visible deformation of blood cells or their dysfunction. It has been shown that the use of sodium oxalate solution with a concentration even lower than isotonic (1.1%) does not induce visible deformations of blood cells [64]. Thus, from the physiological point of view, the concentration of sodium oxalate in the hypothetic [^89^Zr]Zr-oxalate preparation should be in the range of 1.1–1.55% (10.1–15.5 mg/mL) in the absence of additional osmolytes. The estimated (theoretical) lethal dose of sodium oxalate for an adult patient is 15–30 g, but the lowest lethal dose reported is 5 g [65]. Healthy volunteers (women) who received 120 mg of sodium oxalate per day for three days did not have any health problems [66]. For a human, sodium oxalate LD_10_ is 17 mg/kg when administered intravenously [65]. Thus, when 1 mL of [^89^Zr]Zr-oxalate solution with an oxalate ion concentration of 0.1 M is administered to a patient intravenously, the injected amount of oxalate ion will be almost 90 times less than LD_10_ (assuming an average adult weight is 70 kg). These data are also in favor of using 0.1 M oxalic acid to isolate zirconium-89 and, accordingly, in favor of using ZR-resin. Unfortunately, the results of our experiments showed that the concentration of oxalic acid of 0.1 M is insufficient for the complete elution of zirconium-89 from this resin. Another important feature found in the course of this work was that the samples of [^89^Zr]Zr-oxalate preparations, obtained using ZR-resin, exhibit chromatographic behavior differing from [^89^Zr]Zr-oxalate preparations with the same oxalic acid concentration prepared from the origin solution [^89^Zr]Zr-cloride (Dowex).

Implication of Chelex-100 resin after using ZR-resin allows obtaining [^89^Zr]Zr-oxalate preparations with chromatographic behavior of those obtained without using ZR-resin. At the same time, these samples are stable for a long period of time and can withstand raising the pH up to 6.0 ± 0.5. In the course of this work, we also found conditions that make it easy and effective to reconstitute [^89^Zr]Zr-oxalate to [^89^Zr]Zr-citrate using Chelex-100 resin. [^89^Zr]Zr-citrate solutions obtained (especially those in 1 M sodium citrate) are stable enough and are not affected by the increase of pH up to 6.5 ± 0.5, which is convenient for their use in further processing for obtaining radiopharmaceuticals (labeled antibodies). When comparing [^89^Zr]Zr-oxalate and [^89^Zr]Zr-citrate preparations with the same carboxylic acid concentration it can be seen that [^89^Zr]Zr-citrate is less stable than [^89^Zr]Zr-oxalate. However, [^89^Zr]Zr-citrate shelf life is still long enough for possibility of its centralized supply to clinics. Moreover, when comparing the effectiveness of complexation with DFO, [^89^Zr]Zr-citrate showed higher yield than [^89^Zr]Zr-oxalate. Our results are to some extent consistent with the published data on the determination of the stability constants of zirconium complexes. For example, Kobayashi et al. showed that zirconium oxalate complexes have the stability constants of 11.5, 20.8, 27.2 and 29.7 for [Zr(oxalate)]^2+^, [Zr(oxalate)_2_]^0^*_aq._*, [Zr(oxalate)_3_]^2−^ and [Zr(oxalate)_4_]^4−^, respectively [60]. Omar et al. observed the formation of a single complex zirconium-citrate complex with ratio 1:1 [67]. The stability constant of this complex determined by various methods was 7.4–7.6, which is lower than that of zirconium-oxalate complexes. At pH 6.0–7.0, macroscopic Zr^4+^ is known to undergo hydrolysis to the tetranuclear complex [Zr_4_(OH)_8_(OH2)_16_]^8+^ with further polymerization [51]. Therefore, papers on the determination of the stability constants of zirconium complexes where the formation of polynuclear forms is not mentioned cause some concern. On the other hand, in the case of solutions of non-carrier added zirconium radionuclides (microconcentrations), the formation of polynuclear forms is less likely, thus it is acceptable to focus on the published values of the stability constants of mononuclear complexes. It is not clear what causes the difference in the chromatographic behavior and the effectiveness of [^89^Zr]Zr-DFO complex formation. It can be due to the presence of an organic impurity that get into the preparations when using ZR-resin or to the difference in the set of zirconium-89 oxalate complexes in various methods of preparation.

Meijs et al. described the methods for removing oxalic acid from the final solution and evaluated the effect of the method of preparing of radiozirconium solution on the effectiveness of complexation with deferoxamine [46]. With zirconium-89 isolated by hydroxamate method before removal of oxalic acid (the final oxalic acid concentration was 0.2 M), the complexation capacity of deferoxamine was too low to quantitatively form a complex—only about 90% at 10 mM. The authors described lengthy procedures for the production of [^89^Zr]Zr-chloride from purified [^89^Zr]Zr-oxalate involving decarboxylation of the excess oxalic acid with H_2_O_2_ in 6 M HCl at 80 °C followed by drying of the reaction mixture at room temperature *in vacuo* or, alternatively, by the use of room temperature vacuum sublimation techniques at 26.7 mPa. The decarboxylation method gave zirconium-89, which was able to form complexes with a very low yield (<60%) only at very high deferoxamine concentration (>10 mM). The sublimation method resulted in zirconium-89, which formed complexes at very low deferoxamine concentrations (yield > 90% at 10 μM of deferoxamine). Interestingly, zirconium-89 isolated with the anion-exchange method (Dowex) leads to quantitative complexation only at high deferoxamine concentrations. Holland et al. also presented a method of reconstitution [^89^Zr]Zr-oxalate to [^89^Zr]Zr-chloride which is much simpler and more convenient [47]. Zirconium-89 can be removed from oxalic acid solution with 100% efficiency by trapping on an activated a Sep-pak QMA strong anion exchange cartridge. No less than 99.8% of the oxalic acid was then removed by washing the cartridge with a large volume of water, and the activity was eluted with 100% recovery of zirconium-89 with 300–500 μL of 1.0 M HCl(aq.). Standard 0.9% saline can also be used to elute zirconium-89 activity, but the recovery of activity was found to be less efficient (22–38% in 500 μL of saline). The acid can be removed rapidly by boiling the eluate at 110 °C under a continuous stream of argon, then reconstituted in 0.9% saline or 0.1 M HCl(aq.) for further labeling. This method allows to find the effect of the nature of the initial zirconium-89 solution on the complexation efficiency with DOTA [48]. When using [^89^Zr]Zr-oxalate (diluted aliquot of 1.0 M H_2_C_2_O_4_) the complexation with DOTA (99 °C, 120 min, pH 7.0–7.5 (1 M Na_2_CO_3_)) yield was only 65 ± 9.6%, whereas with [^89^Zr]Zr-chloride (90 °C, 45 min, PH 6.9–7.2 (0.5 M HEPES)) it was 100%. Thus, it becomes obvious that methods of preparation of zirconium-89 formulations are able to influence their suitability for further synthesis of radiopharmaceuticals.

A particularly interesting fact discovered during this work is, in our opinion, the demonstrated features of the chromatographic behavior of various zirconium-89 preparations (and, in particular, after reformulation with a QMA-cartridge) and the interrelation of the chromatographic behavior of zirconium-89 preparations and their biodistribution. Undoubtedly, this question needs further, more detailed research. When the chemical form of the zirconium-89 in formulations is not reliably clear, the results of the study of its biodistribution are also very contradictory. For example, according to Holland et al., when [^89^Zr]Zr-chloride is administered intravenously the accumulation of the activity occurs mainly in the liver [47]. The most probable explanation for that is the presence of zirconium-89 in colloidal form. However, in the work of Abou et al., the accumulation of the activity ([^89^Zr]Zr-cloride was administered intravenously as a solution in 0.9% saline, pH 7.2–7.4) was observed in soft tissues, and later on in the skeleton, and the accumulation of the activity in the liver was insignificant [68]. It remains unclear why in one case the hydrolyzed zirconium-89 does not dissolve, and in the other easily goes back to the ionic form. The biodistribution of [^89^Zr]Zr-oxalate is described by Abou et al. as well [68]. The data are similar to our results (Table 4, Sample 1) to a certain degree, taking into account the fact that the parameters of the experiments are not exactly the same. However it can be seen that the pattern of the biodistribution of [^89^Zr]Zr-oxalate preparations in [68] and in Sample 1 (Table 4) is the same: the radioactivity is primarily detected in blood and over time it accumulates in bones. Another example is the comparison of data presented by Bansal et al. [32] with those presented by Abou et al. [68]. According to Abou et al., after the administration of zirconium-89 phosphate within six days, the larger half of the activity was detected in the liver (~60%/g) and the spleen [68]. The data presented by Bansal et al. show that, after the intravenous administration of zirconium-89 phosphate, the larger half of the activity was presented in the liver and the bones [32]. Moreover, seven days post injection, the accumulation in the bones was considerably higher than that in the liver (~60%/g and ~30%/g, respectively). At this stage, it is clear that the use of *Method 1* only (which seems to be very popular) is not enough for a real assessment of the radiochemical purity of zirconium-89 preparations. The method of analysis proposed by us (*Method 2*) gives a more detailed understanding of the speciation of zirconium-89 in the radiopharmaceuticals. Nevertheless, this method also cannot be used independently.

In addition to formulating the final solution of zirconium-89, the effective isolation of zirconium-89 from the irradiated yttrium target is also an important step. We admit the possibility of applying the method described by us to isolate zirconium-89 from the stock solution. The evaluation of the resin suitability for zirconium-89 separation from solutions of an irradiated target was not the goal of this work, and these experiments are in future plans. At the same time, the effectiveness of ZR-resin for the isolation of zirconium-89 from target solutions has already been demonstrated by Graves et al. [50], thus there is no reason to think that the purification in our case can go any other way. We did carry out experiments on metal impurities using model solutions (Y, Cu, and Fe added with the concentration of 25 mg/L) and MP-AES. It was found that with similar model solutions there were no differences in zirconium-89 recovery from Chelex-100 resin and ZR-resin (according to the method prosed in this work). In addition, the results of the quantitative determination of metal impurities showed that using Chelex-100 led to fine (additional) purification from Fe and Cu. Research on this subject are in progress.

## 4. Conclusions

Zirconium itself is a complex chemical element, and its isotope zirconium-89 is a non-conventional radionuclide for nuclear medicine. Considering that, even for radionuclides that are already firmly established in world clinical practice, for example, ^68^Ga, there are still some ambiguities and features in the analysis of radiochemical purity of their preparations [69], much work remains to be done to optimize the methods and approaches to preparation and the quality control (radiochemical purity) of zirconium-89 based formulations.

## 5. Materials and Methods

### 5.1. Chemicals and Reagents

[^89^Zr]ZrCl_4_ in 5 M HCl (20–30 mCi/mL) was purchased from Cyclotron Ltd. (Obninsk, Russia).

Only deionized water 18.2 MΩ·cm (Milli-Q Millipore or TKA Smart2Pure) was used. All chemicals and solvents were of high-purity or pharmaceutical grade (Sigma-Aldrich, Darmstadt, Germany) or Panreac (Barcelona, Spain). Chelex-100 ion exchange resin was purchased from Sigma-Aldrich. ZR-Resin was provided by TrisKem International (Bruz, France). Chromafix 30PS-HCO_3_ cartridges were purchased from Macherey-Nagel (Düren, Germany). Deferoxamine was purchased from Sigma-Aldrich (Darmstadt, Germany).

### 5.2. Measurement of Radioactivity

Measurement of zirconium-89 absolute activity was performed using Atomlab^TM^ 500 Dose Calibrator (Biodex, Shirley, NY, USA) and RIS-A1 (Amplituda Scientific and Technical Center, Moscow, Russia) dose calibrator. Relative activity (count rate) of the samples was measured by RFT 20,046 γ-counter (Veb Robotron-Messelektronik, Dresden; detector: NaI crystal), as well as with Wizard^2^ 2480 automatic γ-counter (PerkinElmer, Waltham, MA, USA).

### 5.3. Distribution Coefficients

The mass distribution coefficient *D_g_* was measured by batch (static) method. A known amount of the ion exchanger and an aliquot of the zirconium-89 solution (10 μL) were added to a solution of known volume; this was carried out using 5 mL Eppendorf centrifuge tubes. The samples were mixed in the mechanical mixer with temperature control Bioer Mixing block MB-102 (Bioer, Hangzhou, China) for 5, 10, 20, 30, and 60 min at 20 °C with stirring speed of 1350 rpm. The samples were centrifuged (Heidolph, Germany) for 1 min at 15,000 rpm. After 1 min, the tubes were removed carefully (to avoid phase mixing). Then, the aliquots (100 μL) of every solution were taken and their activities were measured.

The mass distribution coefficient was defined according to the following equation (time point 30 min):

(1) $$D_{g}=\frac{A_{0}-A}{A}\times \frac{V\left(mL\right)}{m\left(g\right)}$$

where *A_0_* is the count rate of the sample aliquot before the contact with the resin (decay corrected); *A* is the count rate of the sample aliquot after the contact with the resin (decay corrected); *V* is the solution volume, mL (5 mL); and *m* is the weight of dry resin, *g* (0.1 g). The value of *A*_0_ does not require correction to the resin swelling due to the large value of the $\frac{V\left(mL\right)}{m\left(g\right)}$ ratio.

### 5.4. Dynamic Studies

To study zirconium-89 ion exchange behavior in dynamic conditions, polyetheretherketone (PEEK) chromatographic columns of 50 mm × 2.1 mm diameter and 100 mm × 2.1 mm diameter (VICI Jour, Schenkon, Switzerland) and 1 mL polypropylene cartridges with polyethylene frits (Supelco Darmstadt, Germany) were used. Transfer of the working solutions was performed by syringes manually or with a syringe pump (flow rate, 1 mL/min). The amount of each tested resin was 60 mg.

### 5.5. TLC

Quality control of the preparations was carried out with radio-TLC chromatography (PET-MiniGita radio-TLC scanner). Several different chromatographic systems were used for the analysis of the samples. In most cases, silica gel impregnated fiberglass strips (iTLC-SG Fisher Scientific, Hampton, NH, USA) were used as a stationary phase (iTLC-SG/50 mM DTPA pH 7.0—*Method 1*; and iTLC-SG/CH_3_OH-H_2_O (1:1), 4% TFA (*v*/*v*)—*Method 2*). Other system was iTLC-SA/50 mM EDTA (pH 5).

### 5.6. Biodistribution Experiments

All experiments involving animals were performed following the ethical standards, Russian animal protection laws and guidelines for scientific animal trials [GOST 33216-2014 Guidelines for accommodation and care of animals. Species-specific provisions for laboratory rodents and rabbits] with permission of Ethical board of State Research Center—Burnasyan Federal Medical Biophysical Center of Federal Medical Biological Agency (decision No. 86 10.11.2017).

Experiments were carried out in healthy BALB/C mice. [^89^Zr]Zr-oxalate or [^89^Zr]Zr-citrate solutions (100 μL, 50–100 µCi) were injected into the tail vein. For ex vivo biodistribution experiments, animals were euthanized by decapitation 1 and 24 h after injection of zirconium-89 preparations. At each time point of each sample, 3 animals were taken. Tissues were extracted, washed, weighed and measured with Wizard^2^ 2480 automatic γ-counter (PerkinElmer). For in vivo biodistribution experiments, animals were anesthetized under 1–2% isoflurane 24 h after injection of zirconium-89 preparations and underwent PET imaging using a small animal PET/X-RAY system (Genesys4, Sofie BioSystems, Culver City, CA, USA).

## Figures and Tables

**Table 1:** Radio-chromatograms of different zirconium-89 preparations with iTLC-SG/CH_3_OH-H_2_O (1:1), 4% TFA (*v*/*v*) system (*Method 2*).

[^89^Zr]Zr-Oxalate0.1 M Oxalic Acid	[^89^Zr]Zr-Oxalate 0.1 M Oxalic Acid (Neutralized to pH~7)	[^89^Zr]Zr-Oxalate1.0 M Oxalic Acid	[^89^Zr]ZrCl_4_ in 1 M HCl	[^89^Zr]Zr in 0.1 M HCl
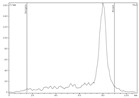	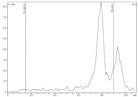	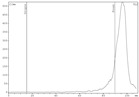	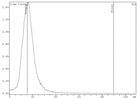	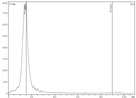

**Table 2:** Efficiency of zirconium-89 elution from ZR-resin with different eluents.

Eluent	Efficiency of [^89^Zr]Zr Elution from ZR-Resin, %
Oxalic acid 1.0 M	99.0 ± 1.0
Oxalic acid 0.5 M	95.2 ± 1.8
Oxalic acid 0.1 M	90.1 ± 4.9
Sodium oxalate 0.1 M	~40
Citric acid 0.1 M	<1
Citric buffer 0.1 M (pH 4.0)	~2
Malonic acid 0.1 M	<1
Succinic acid 0.1 M	<1

**Table 3:** The main characteristics of the proposed processes for formulating zirconium-89 solutions for radiopharmaceutical preparations.

Formulation Method	Total Yield of ^89^Zr, %	pH_max._	Shelf Life
ZR-resin only (0.1 M oxalic acid)	90 ± 2%	5.0 ± 0.5	8–9 days
ZR-resin + Chelex-100 (0.1 M sodium oxalate)	93 ± 2%	6.0 ± 0.5	>10 days
ZR-resin + Chelex-100 (0.1 M sodium citrate)	90 ± 1%	5.0 ± 0.5	3–4 days
ZR-resin + Chelex-100 (1.0 M sodium citrate)	94 ± 2%	6.5 ± 0.5	6–7 days

**Table 4:** Relation between chromatographic behavior of [^89^Zr]Zr-oxalate preparations and their biodistribution evaluated using ex vivo direct radiometry.

**Method**	**[^89^Zr]Zr-Oxalate** **Sample 1**		**[^89^Zr]Zr-Oxalate** **Sample 2**		**[^89^Zr]Zr-Oxalate** **Sample 3**	
	**pH 5.5**		**pH 7.0**		**pH 9.0**	
**Chromatography (Radio-TLC)**						
iTLC-SG/50 mM DTPA (pH 7.0) *Method 1*	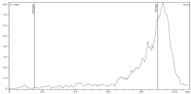		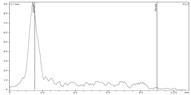		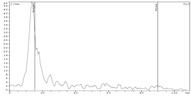	
	RCP ~ 100%		RCP ~ 0%		RCP ~ 0%	
iTLC-SG/CH_3_OH-H_2_O (1:1), 4% TFA (*v*/*v*) *Method 2*	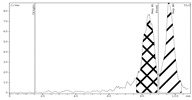		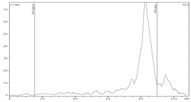		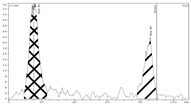	
	RCP ~ 100%		RCP ~ 100%		RCP < 30%	
**Biodistribution (ID/g, %)**						
**Organ/Tissue**	**Time after Intravenous Administration**					
	**1 h**	**24 h**	**1 h**	**24 h**	**1 h**	**24 h**
blood	3.57 ± 0.61	2.19 ± 0.30	3.36 ± 0.60	2.15 ± 0.19	0.99 ± 0.12	0.13 ± 0.02
lungs	1.22 ± 0.29	1.14 ± 0.19	1.54 ± 0.35	1.12 ± 0.09	1.05 ± 0.30	0.17 ± 0.08
heart	1.70 ± 0.16	1.09 ± 0.14	1.42 ± 0.39	1.06 ± 0.11	0.05 ± 0.01	0.11 ± 0.03
stomach	0.47 ± 0.16	0.30 ± 0.05	0.39 ± 0.01	0.25 ± 0.02	0.27 ± 0.07	0.04 ± 0.01
liver	0.68 ± 0.20	0.93 ± 0.05	0.77 ± 0.19	0.88 ± 0.07	5.12 ± 1.81	4.88 ± 1.07
kidney	0.59 ± 0.11	1.52 ± 0.24	0.65 ± 0.16	1.19 ± 0.82	0.27 ± 0.11	0.60 ± 0.29
bladder	0.21 ± 0.01	0.32 ± 0.17	0.34 ± 0.11	0.48 ± 0.22	0.11 ± 0.06	0.06 ± 0.02
s. intestine	0.47 ± 0.02	0.42 ± 0.07	0.52 ± 0.15	0.39 ± 0.06	0.22 ± 0.07	0.06 ± 0.02
l. intestine	0.18 ± 0.01	0.55 ± 0.14	0.22 ± 0.15	0.38 ± 0.11	0.06 ± 0.03	0.10 ± 0.01
muscle	0.11 ± 0.05	0.11 ± 0.01	0.22 ± 0.03	0.14 ± 0.03	0.05 ± 0.01	0.02 ± 0.01
femur	0.26 ± 0.08	0.95 ± 0.25	0.37 ± 0.27	0.97 ± 0.17	0.14 ± 0.02	0.27 ± 0.04

**Table 5:** Radio-TLC chromatograms of representative [^89^Zr]Zr-oxalate samples (prepared for in vivo μPET imaging (Figure 5) obtained using both methods.

**Method**	**[^89^Zr]Zr-Oxalate Sample 1**	**[^89^Zr]Zr-Oxalate Sample 2**
iTLC-SG/50 mM DTPA(pH 7.0) *Method 1*	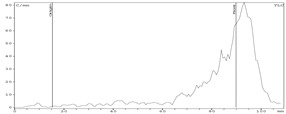	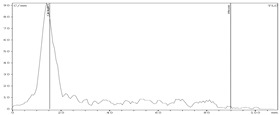
	RCP ~ 100%	RCP ~ 0%
iTLC-SG/CH_3_OH-H_2_O (1:1), 4% TFA (*v*/*v*) *Method 2*	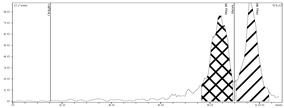 RCP ~ 100%	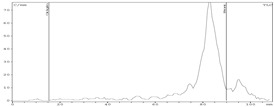 RCP ~ 100%
	**[^89^Zr]Zr-Oxalate Sample 3**	**[^89^Zr]Zr-Oxalate Sample 4**
iTLC-SG/50 mM DTPA (pH 7.0) *Method 1*	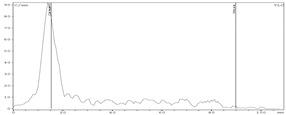	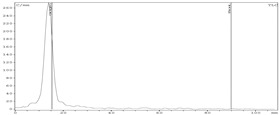
	RCP ~ 0%	RCP ~ 0%
iTLC-SG/CH_3_OH-H_2_O (1:1), 4% TFA (*v*/*v*) *Method 2*	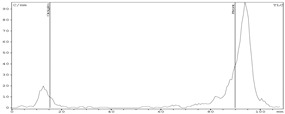	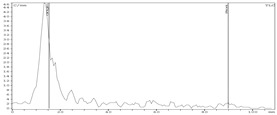
	RCP ~ 90%	RCP ~ 0%

## Supplementary Materials

The following are available online, Table S1: Radio-chromatograms of different zirconium-89 – oxalic acid preparations with iTLC-SG/CH_3_OH-H_2_O (1:1), 4% TFA (*v*/*v*) system. Table S2: Radio-chromatograms of different zirconium-89 – HCl preparations with iTLC-SG/CH3OH-H2O (1:1), 4% TFA (*v*/*v*) system. Figure S1: Radio-chromatograms of different zirconium-89 preparations in HCl (iTLC-SG/50 mM DTPA pH 7.0). Figure S2: Elution profiles of ZR-resin column (50 mg, 50 mm × Ø2.1 mm) with oxalic acid of different concentrations. Table S3: Radio-TLC analysis results of [^89^Zr]Zr-oxalate preparations obtained with ZR-resin and solutions of different oxalic acid concentrations. Table S4: Radio-TLC analysis results for [^89^Zr]Zr-oxalate preparations obtained without and after ZR-resin. Figure S3: Chelex-100 column elution profile using 0.1 M Na_3_Citr and 0.1 M Na_2_C_2_O_4_ as eluents. Figure S4: The effect of various solutions used for column wash on recovery efficiency of zirconium-89 with Chelex-100 resin. Table S5: Radio-chromatograms of different [^89^Zr]Zr-citrate preparations. Figure S5: The scheme of methods proposed for preparation of zirconium-89 solutions for radiopharmaceutical purposes. Table S6: Radio-TLC analysis results of [^89^Zr]Zr-oxalate preparations during their storage. Table S7: Examples of analysis of the products of zirconium-89 reactions with DFO. Table S8: The results of the analysis of various preparations of zirconium-89 while keeping the samples at room temperature and when heated. Figure S6: The scheme of zirconium-89 reconstitution from oxalic acid solution into hydrochloric acid solution using Chromafix 30PS-HCO_3_ anion-exchange cartridge. Table S9: Radio-TLC results (method 2) of zirconium-89 samples obtained in 1.0 M HCl using oxalate-free technology and using the reconstitution method from oxalic acid solution. Table S10: Radio-chromatograms in various systems. Zirconium-89 samples obtained in 1.0 M HCl using oxalate-free technology and those reconstituted from oxalic acid solution (pH = 6).

## References

[1] Knapp F.F., Dash A. (2016). Introduction: Radiopharmaceuticals Play an Important Role in Both Diagnostic and Therapeutic Nuclear Medicine. Radiopharmaceuticals for Therapy.

[2] Qin Z. (2015). Recent advances of injectable radiopharmaceuticals for nuclear imaging and therapy: A new era in nuclear medicine. Mater. Technol..

[3] Basu S., Alavi A. (2016). PET-Based Molecular Imaging in Evolving Personalized Management Design. PET Clin..

[4] Vasdev N., Alavi A. (2017). Novel PET Radiotracers with Potential Clinical Applications. PET Clin..

[5] Velikyan I. (2014). Prospective of 68Ga-Radiopharmaceutical Development. Theranostics.

[6] Jackson I.M., Scott P.J., Thompson S. (2017). Clinical Applications of Radiolabeled Peptides for PET. Semin. Nucl. Med..

[7] Banerjee S.R., Pomper M.G. (2013). Clinical applications of Gallium-68. Appl. Radiat. Isot..

[8] Brasse D., Nonat A. (2015). Radiometals: Towards a new success story in nuclear imaging?. Dalton Trans..

[9] Krasikova R.N., Aliev R.A., Kalmykov S.N. (2016). The next generation of positron emission tomography radiopharmaceuticals labeled with non-conventional radionuclides. Mendeleev Commun..

[10] Gourni E., Henriksen G. (2017). Metal-Based PSMA Radioligands. Molecules.

[11] Price E.W., Orvig C. (2014). Matching chelators to radiometals for radiopharmaceuticals. Chem. Soc. Rev..

[12] Olivier K.J., Hurvitz S.A. (2016). Antibody-Drug Conjugates: Fundamentals, Drug Development, and Clinical Outcomes to Target Cancer.

[13] Braad P.E.N., Hansen S.B., Thisgaard H., Høilund-Carlsen P.F. (2015). PET imaging with the non-pure positron emitters: (55)Co, (86)Y and (124)I. Phys. Med. Biol..

[14] Lopez-Rodriguez V., Gaspar-Carcamo R.E., Pedraza-Lopez M., Rojas-Calderon E.L., de Murphy C.A., Ferro-Flores G., Avila-Rodriguez M.A. (2015). Preparation and preclinical evaluation of 66Ga-DOTA-E(c(RGDfK))2 as a potential theranostic radiopharmaceutical. Nucl. Med. Biol..

[15] Hernandez R., Valdovinos H.F., Yang Y., Chakravarty R., Hong H., Barnhart T.E., Cai W. (2014). ^44^Sc: An Attractive Isotope for Peptide-Based PET Imaging. Mol. Pharm..

[16] Asabella A.N. (2014). The Copper Radioisotopes: A Systematic Review with Special Interest to ^64^Cu. Biomed. Res. Int..

[17] Laboratoire National Henri Becquerel/CEA (2017). ^89^Zr. Table de Radionucleides (Recommended data).

[18] Fischer G., Seibold U., Schirrmacher R., Wängler B., Wängler C. (2013). (89)Zr, a radiometal nuclide with high potential for molecular imaging with PET: Chemistry, applications and remaining challenges. Molecules.

[19] Coursey J.S., Schwab D.J., Tsai J.J., Dragoset R.A. (2018). Atomic Weights and Isotopic Compositions with Relative Atomic Masses (web). NIST Physical Measurement Laboratory.

[20] Van Dongen G.A., Visser G.W., Lub-de Hooge M.N., De Vries E.G., Perk L.R. (2007). Immuno-PET: A navigator in monoclonal antibody development and applications. Oncologist.

[21] Sharifian M., Sadeghi M., Alirezapour B. (2017). Utilization of GEANT to calculation of production yield for ^89^Zr by charge particles interaction on ^89^Y, ^nat^Zr and ^nat^Sr. Appl. Rad. Isot..

[22] Kandil S.A., Spahn I., Scholten B., Saleh Z.A., Saad S.M.M., Coenen H.H., Qaim S.M. (2007). Excitation function of (α,xn) reactions on ^nat^Rb and ^nat^Sr from threshold to 26 MeV: Possibility of ^87^Y, ^88^Y and ^89^Zr. Appl. Radiat. Isot..

[23] Zweit J., Downey S., Sharma H.L. (1991). Production of no-carrier-added zirconium-89 for positron emission tomography. Int. J. Rad. Appl. Instrum. A.

[24] Omara H.M., Hassan K.F., Kandil S.A., Hegazy F.E., Saleh Z.A. (2009). Proton induced reactions on 89Y with particular reference to the production of the medically interesting radionuclide ^89^Zr. Radiochim. acta.

[25] Kasbollah A., Eu P., Cowell S., Deb P. (2013). Review on production of 89Zr in a medical cyclotron for PET radiopharmaceuticals. J. Nucl. Med. Technol..

[26] Synowiecki M.A., Perk L.R., Nijsen J.F.W. (2018). Production of novel diagnostic radionuclides in small medical cyclotrons. EJNMMI Radiopharm. Chem..

[27] Link J.M., Krohn K.A., O’Hara M.J. (2017). A simple thick target for production of ^89^Zr using an 11 MeV cyclotron. Appl. Radiat. Isot..

[28] Deri M.A., Zeglis B.M., Francesconi L.C., Lewis J.S. (2013). PET imaging with ^89^Zr: From radiochemistry to the clinic. Nucl. Med. Biol..

[29] Vugts D.J., Visser G.W., van Dongen G.A. (2013). ^89^Zr-PET Radiochemistry in the Development and Application of Therapeutic Monoclonal Antibodies and Other Biologicals. Curr. Top. Med. Chem..

[30] Mealey J. (1957). Turn-over of carrier-free zirconium-89 in Man. Nature.

[31] Sato N., Wu H., Asiedu K.O., Szajek L.P., Griffiths G.L., Choyke P.L. (2015). ^89^Zr-oxine complex PET cell imaging in monitoring cell-based therapies. Radiology.

[32] Bansal A., Pandey M.K., Demirhan Y.E., Nesbitt J.J., Crespo-Diaz R.J., Terzic A., Behfar A., DeGrado T.R. (2015). Novel ^89^Zr cell labeling approach for PET-based cell trafficking studies. EJNMMI Res..

[33] Asiedu K.O., Koyasu S., Szajek L.P., Choyke P.L., Sato N. (2017). Bone Marrow Cell Trafficking Analyzed by ^89^Zr-oxine Positron Emission Tomography in a Murine Transplantation Model. Clin. Cancer Res..

[34] Park J.A., Lee Y.J., Lee J.W., Yoo R.J., Shin U.C., Lee K.C., Kim B.I., Kim K.M., Kim J.Y. (2016). Evaluation of [^89^Zr]-Oxalate as a PET Tracer in Inflammation, Tumor, and Rheumatoid Arthritis Models. Mol. Pharm..

[35] Van de Watering F.C., Rijpkema M., Perk L., Brinkmann U., Oyen W.J., Boerman O.C. (2014). Zirconium-89 labeled antibodies: A new tool for molecular imaging in cancer patients. Biomed. Res. Int..

[36] Jauw Y.W., Hoekstra O.S., Hendrikse N.H., Vugts D.J., Zijlstra J.M., Huisman M.C., van Dongen G.A. (2016). Immuno-Positron Emission Tomography with Zirconium-89-Labeled Monoclonal Antibodies in Oncology: What Can We Learn from Initial Clinical Trials?. Front. Pharmacol..

[37] Das S.S., Chattopadhyay S., Barua L., Alam M.N., Kumar U. (2017). Production and radiochemical separation of a potential immuno-PET imaging agent ^89^Zr from proton irradiated ^nat^Y target. J. Radioanal. Nucl. Chem..

[38] Kandil S.A., Scholten B., Saleh Z.A., Youssef A.M., Qaim S.M., Coenen H.H. (2007). A comparative study on the separation of radiozirconium via ion-exchange and solvent extraction techniques, with particular reference to the production of ^88^Zr and ^89^Zr in proton induced reactions on yttrium. J. Radioanal. Nucl. Chem..

[39] Dutta B., Maiti M., Lahiri S. (2009). Production of ^88,89^Zr by proton induced activation of natY and separation by SLX and LLX. J. Radioanal. Nucl. Chem..

[40] Tang Y., Li S., Yang Y., Chen W., Wei H., Wang G., Yang J., Liao J., Luo S., Liu N. (2016). A simple and convenient method for production of ^89^Zr with high purity. Appl. Radiat. Isot..

[41] Link J.M., Krohn K.A., Eary J.F., Kishore R., Lewellen T.K., Johnson M.W., Badger C.C., Richter K.Y., Nelp W.B.J. (1986). ^89^Zr for antibody labeling and positron emission tomograph. Labeled Compd. Radiopharm..

[42] Dejesus O.T., Nickles R.J. (1990). Production and purification of 89Zr, a potential PET antibody label. Int. J. Rad. Appl. Instrum. A.

[43] Baroncelli F., Grossi G. (1965). The complexing power of hydroxamic acids and its effect on the behaviour of organic extractants in the reprocessing of irradiated fuels—The complexes between benzohydroxamic acid and zirconium, iron (III) and uranium (VI). J. Inorg. Nucl. Chem..

[44] Fadeeva V.I., Tikhomirova T.I., Yuferova I.B., Kudryavtsev G.V. (1989). Preparation. properties and analytical application of silica with chemically grafted hydroxamic acid groups. Anal. Chim. Acta.

[45] Phillips R.J., Fritz J.S. (1982). Extraction of metal ions by N-phenyl-, N-methyl-, and N-unsubstituted hydroxamic acid resins. Anal. Chim. Acta.

[46] Meijs W.E., Herscheid J.D., Haisma H.J., Wijbrandts R., van Langevelde F., Van Leuffen P.J., Mooy R., Pinedo H.M. (1994). Production of Highly Pure No-carrier Added ^89^Zr for the Labelling of Antibodies with a Positron Emitter. Appl. Radiat. Isot..

[47] Holland J.P., Sheh Y., Lewis J.S. (2009). Standardized methods for the production of high specific-activity zirconium-89. Nucl. Med. Biol..

[48] Pandya D.N., Bhatt N., Yuan H., Day C.S., Ehrmann B.M., Wright M., Bierbach U., Wadas T.J. (2017). Zirconium tetraazamacrocycle complexes display extraordinary stability and provide a new strategy for zirconium-89-based radiopharmaceutical development. Chem. Sci..

[49] Bombard A. “ZR Resin,” Triskem Infos, Vol. 15. http://www.triskem-international.com/scripts/files/59d1f4fc31f796.50370140/tki_15_en_web.pdf.

[50] Graves S.A., Kutyreff C., Barrett K.E., Hernandez R., Ellison P.A., Happel S., Aluicio-Sarduy E., Barnhart T.E., Nickles R.J., Engle J.W. (2018). Evaluation of a chloride-based ^89^Zr isolation strategy using a tributylphosphate (TBP)-functionalized extraction resin. Nucl. Med. Biol..

[51] Solovkin A.S., Tsvetkova Z.N. (1962). The chemistry of aqueous solutions of zirconium salts (Does the zirconyl ion exist?). Russ. Chem. Rev..

[52] El-Sweify F.H., Shabana R., Abdel-Rahman N., Aly H.F. (1985). Distribution of some Actinides and Fission Products between the Chelating Ion Exchanger Chelex-100 and Certain Carboxylic Acid Solutions. Radiochim. Acta.

[53] Zeglis B.M., Lewis J.S. (2015). The bioconjugation and radiosynthesis of ^89^Zr-DFO-labeled antibodies. J. Vis. Exp..

[54] Li N., Yu Z., Pham T.T., Blower P.J., Yan R. (2017). A generic ^89^Zr labeling method to quantify the in vivo pharmacokinetics of liposomal nanoparticles with positron emission tomography. Int. J. Nanomedicine.

[55] Bhatt N.B., Pandya D.N., Xu J., Tatum D., Magda D., Wadas T.J. (2017). Evaluation of macrocyclic hydroxyisophthalamide ligands as chelators for zirconium-89. PLoS ONE.

[56] Tinianow J.N., Pandya D.N., Pailloux S.L., Ogasawara A., Vanderbilt A.N., Gill H.S., Williams S.P., Wadas T.J., Magda D., Marik J. (2016). Evaluation of a 3-hydroxypyridin-2-one (2, 3-HOPO) based macrocyclic chelator for ^89^Zr^4+^ and its use for immunoPET imaging of HER2 positive model of ovarian carcinoma in mice. Theranostics.

[57] Dias G.M. (2017). Evaluation of Cyclotron Produced Radiometals for Radiolabeling of Immuno-and Bio-Conjugates for Nuclear Imaging. Ph.D. Thesis.

[58] Ulmert D., Evans M.J., Holland J.P., Rice S.L., Wongvipat J., Pettersson K., Lewis J.S. (2012). Imaging androgen receptor signaling with a radiotracer targeting free prostate-specific antigen. Cancer Discov..

[59] Friend M.T., Wall N.A. (2019). Stability constants for Zirconium(IV) complexes with EDTA, CDTA, and DTPA in perchloric acid solutions. Inorg. Chim. Acta.

[60] Kobayashi T., Sasaki T., Takagi I., Moriyama H. (2009). Zirconium solubility in ternary aqueous system of Zr(IV)-OH-carboxylates. J. Nucl. Sci. Technol..

[61] O’Hara M.J., Murray N.J., Carter J.C., Morrison S.S. (2018). Optimized anion exchange column isolation of zirconium-89 from yttrium cyclotron target: Method development and implementation on an automated fluidic platform. J. Chromatogr. A.

[62] Leendertz G., Gromelski B. (1922). Zwei neue Methoden zur Fibrinogenbestimmung. Arch. Exp. Path. u Pharmakol..

[63] Hitchcock D.I., Dougan R.B. (1935). Freezing points of anti-coagulant salt solutions. J. Gen. Physiol..

[64] Sasano K.T., Ordway W.H., Medlar E.M. (1936). A study of certain factors which influence the sedimentation rates of erythrocytes with especial emphasis upon the effect of temperature. Am. J. Clin. Pathol..

[65] Von Burg R. (1994). Toxicology Update. Oxalic acid and Sodium oxalate. J. Appl. Toxicol..

[66] Hanson C.F., Frankos V.H., Thompson W.O. (1989). Bioavailability of oxalic acid from spinach, sugar beet fibre and a solution of sodium oxalate consumed by female volunteers. Food Chem. Toxicol..

[67] Omar A., Ali E. (2015). Stability constants and stoichiometries of chromium and zirconium carboxylates complexes calculated by four comparative methods. Int. J. Adv. Chem..

[68] Abou D.S., Ku T., Smith-Jones P.M. (2011). In vivo biodistribution and accumulation of ^89^Zr in mice. Nucl. Med. Biol..

[69] Larenkov A.A., Maruk A.Y., Kodina G.E. (2018). Intricacies of the Determination of the Radiochemical Purity of 68Ga Preparations: Possibility of Sorption of Ionic ^68^Ga Species on Reversed-Phase Columns. Radiochemistry.

