# Supplementary material for: Preparation of Zirconium-89 Solutions for Radiopharmaceutical Purposes: Interrelation Between Formulation, Radiochemical Purity, Stability and Biodistribution
Source: Molecules. 2019 Apr 18;24(8):1534. doi: 10.3390/molecules24081534 (PMC6514948; doi:10.3390/molecules24081534)
Supplement: Supplementary file 1 [file molecules-24-01534-s001.pdf]

molecules

Supplementary materials for:

Preparation of Zirconium-89 Solutions for Radiopharmaceutical Purposes: Interrelation Between Formulation, Radiochemical Purity, Stability and Biodistribution

Anton Larenkov *, Victor Bubenschikov, Artur Makichyan, Maria Zhukova, Alina Krasnoperova, Galina Kodina

Content:

Table S1. Radio-chromatograms of different zirconium-89 – oxalic acid preparations with iTLC-SG / CH_3OH-H_2O (1:1), 4% TFA (v/v) system.. 2

Table S2. Radio-chromatograms of different zirconium-89 – HCl preparations with iTLC-SG / CH3OH-H2O (1:1), 4% TFA (v/v) system.. 2

Figure S1. Radio-chromatograms of different zirconium-89 preparations in HCl (iTLC-SG / 50 mM DTPA pH 7.0)... 2

Figure S2. Elution profiles of ZR-resin column (50 mg, 50 mm × Ø2.1 mm) with oxalic acid of different concentrations.. 3

Table S3. Radio-TLC analysis results of [^{89}Zr]Zr-oxalate preparations obtained with ZR-resin and solutions of different oxalic acid concentrations.. 3

Table S4. Radio-TLC analysis results for [^{89}Zr]Zr-oxalate preparations obtained without and after ZR-resin.. 4

Figure S3. Chelex-100 column elution profile using 0,1 M Na_3Citr and 0.1 M $Na_2C_2O_4$ as eluents..... 4

Figure S4. The effect of various solutions used for column wash on recovery efficiency of zirconium-89 with Chelex-100 resin... 5

Table S5. Radio-chromatograms of different [^{89}Zr]Zr-citrate preparations................................ 5

Figure S5. The scheme of methods proposed for preparation of zirconium-89 solutions for radiopharmaceutical purposes.. 6

Table S6. Radio-TLC analysis results of [^{89}Zr]Zr-oxalate preparations during their storage........... 7

Table S7. Examples of analysis of the products of zirconium-89 reactions with DFO.................... 8

Table S8. The results of the analysis of various preparations of zirconium-89 while keeping the samples at room temperature and when heated.. 9

Figure S6. The scheme of zirconium-89 reconstitution from oxalic acid solution into hydrochloric acid solution using Chromafix 30PS-HCO_3 anion-exchange cartridge......................... 10

Table S9. Radio-TLC results (method 2) of zirconium-89 samples obtained in 1.0 M HCl using oxalate-free technology and using the reconstitution method from oxalic acid solution... 10

Table S10. Radio-chromatograms in various systems. Zirconium-89 samples obtained in 1.0 M HCl using oxalate-free technology and those reconstituted from oxalic acid solution........... 10

Table S1. Radio-chromatograms of different zirconium-89 – oxalic acid preparations with iTLC-SG / CH_3OH-H_2O (1:1), 4% TFA (v/v) system.

$[^{89}Zr]$Zr-oxalate 0.1 M oxalic acid	$[^{89}Zr]$Zr-oxalate 0.1 M oxalic acid (neutralized to pH~7)	$[^{89}Zr]$Zr-oxalate 0.27 M oxalic acid	$[^{89}Zr]$Zr-oxalate 0.27 M oxalic acid (neutralized to pH ~7)	$[^{89}Zr]$Zr-oxalate 1.0 M oxalic acid
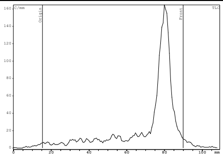	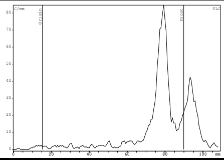	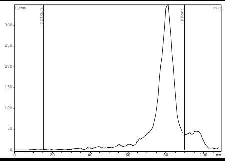	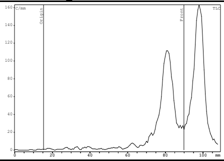	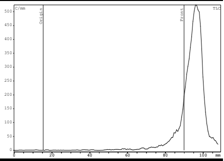

Table S2. Radio-chromatograms of different zirconium-89 – HCl preparations with iTLC-SG / CH3OH-H2O (1:1), 4% TFA (v/v) system.

$[^{89}Zr]ZrCl_4$ in 5 M HCl	$[^{89}Zr]ZrCl_4$ in 1 M HCl	$[^{89}Zr]$Zr in 0.1 M HCl	$[^{89}Zr]$Zr in 0.1 M HCl (neutralized to pH 7)
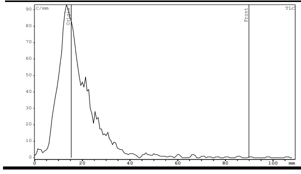	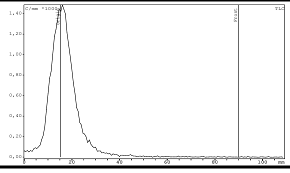	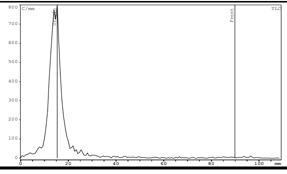	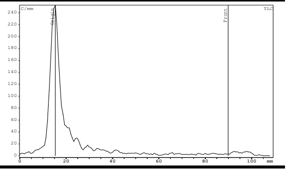

These samples were prepared from $[^{89}Zr]ZrCl_4$ in 5 M HCl initial solution obtained by the manufacturer using oxalate-free method (with Dowex 1×8 anion-exchange resin)

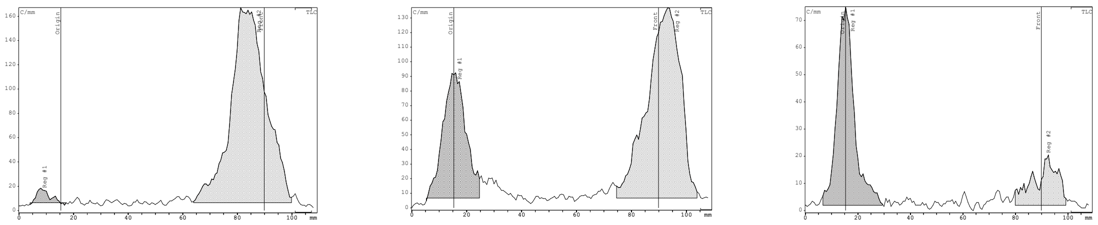

(a) (b) (c)

Figure S1. Radio-chromatograms of different zirconium-89 preparations in HCl (iTLC-SG / 50 mM DTPA pH 7.0): (a) 5 M HCl; (b) 1 M HCl; (c) 0.1 M HCl.

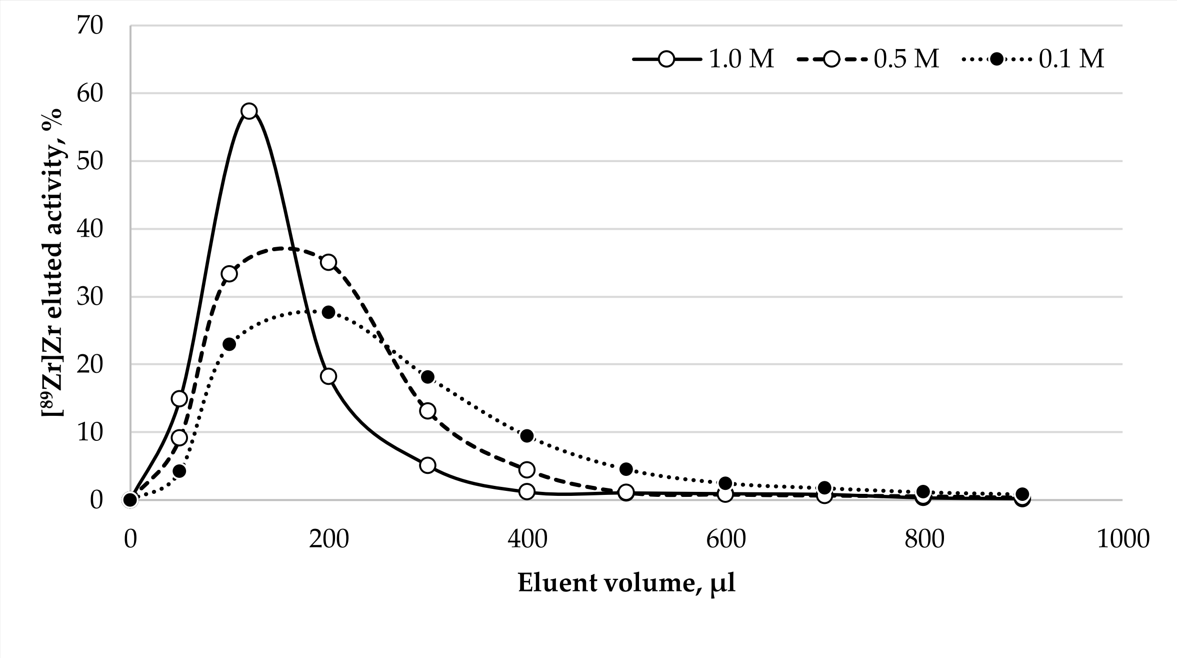

Figure S2. Elution profiles of ZR-resin column (50 mg, 50 mm × Ø2.1 mm) with oxalic acid of different concentrations.

Table S3. Radio-TLC analysis results of [^{89}Zr]Zr-oxalate preparations obtained with ZR-resin and solutions of different oxalic acid concentrations (final pH of all preparations was 6.5).

	**Oxalic acid concentration in the sample (moles/L)**				
	**0.1**	**0.2**	**0.3**	**0.4**	**0.5**
method 1	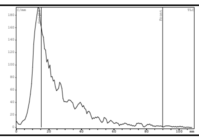	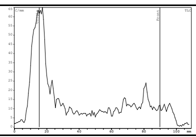	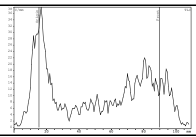	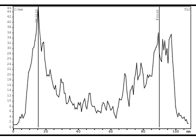	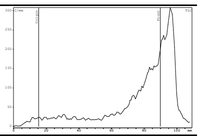
%§	100	~55	~40	~35	~10
method 2	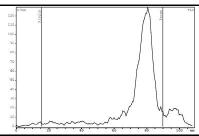	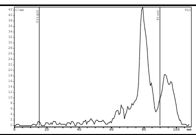	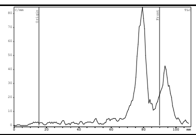	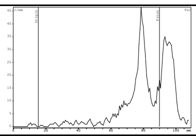	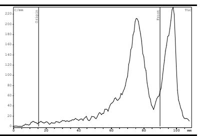
%§§	~10	~30	~35	~45	~50

§ % of zirconium-89 activity at the origin of chromatogram; §§ % of zirconium-89 activity at R*f* 1.0 peak.

Table S4. Radio-TLC analysis results for [^{89}Zr]Zr-oxalate preparations obtained without and after ZR-resin.

	**[^{89}Zr]Zr-oxalate in 0.1 M oxalic acid (without ZR-resin)**					
pH	1	3	5	6	7	8
Method 1	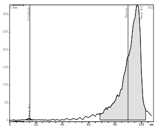	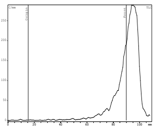	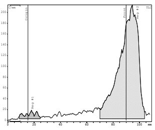	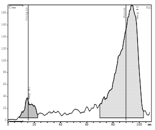	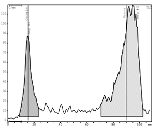	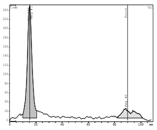
Method 2	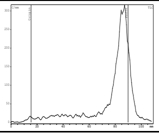	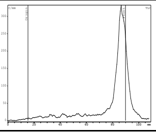	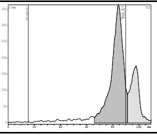	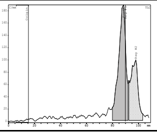	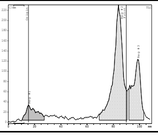	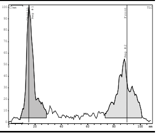
	**[^{89}Zr]Zr-oxalate in 0.1 M oxalic acid (after ZR-resin)**					
pH	1	3	5	6	7	8
Method 1	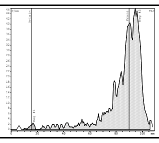	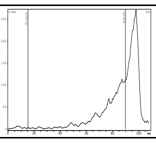	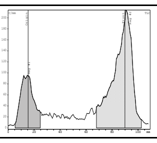	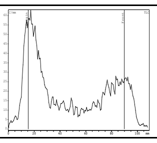	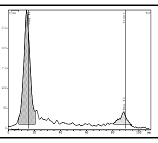	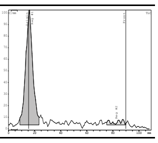
Method 2	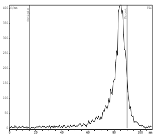	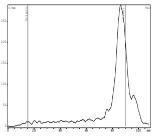	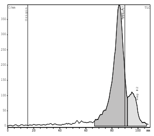	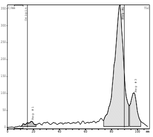	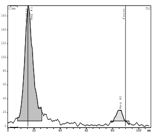	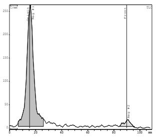

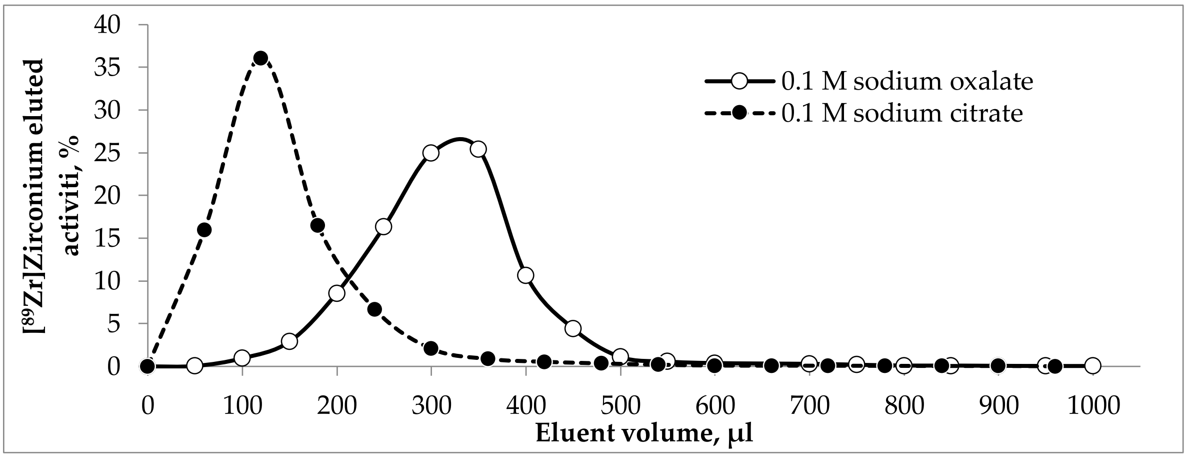

Figure S3. Chelex-100 column elution profile using 0,1 M Na_3Citr and 0.1 M $Na_2C_2O_4$ as eluents (50 mm ×Ø2.1 mm column, 60 mg of resin, load from 0.5 M oxalic acid medium (ZR-resin), eluent flow rate – 1 mL/min).

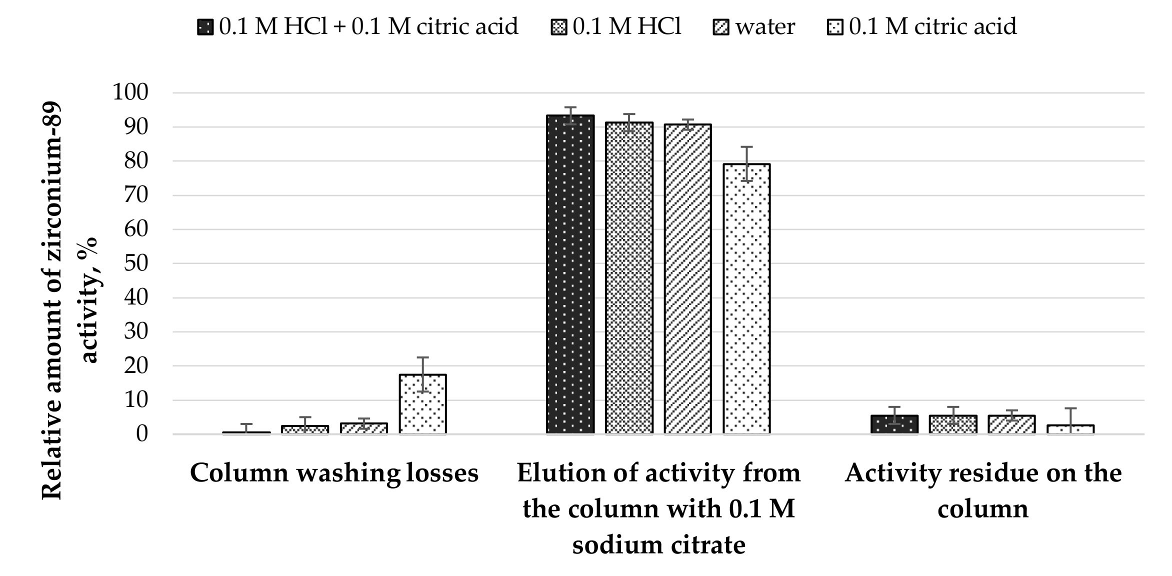

Figure S4. The effect of various solutions used for column wash on recovery efficiency of zirconium-89 with Chelex-100 resin (50 mm ×Ø2.1 mm column, 60 mg of resin, load from 0.5 M oxalic acid medium (ZR-resin), losses at the sorption step – 0.7 %, eluents flow rate – 1 mL/min).

Table S5. Radio-chromatograms of different [^{89}Zr]Zr-citrate preparations.

	**pH**			
	**4.5**		**6.5**	
	Method 1	*Method 2*	*Method 1*	*Method 2*
**[^{89}Zr]Zr-citrate in 0.1M citric acid**	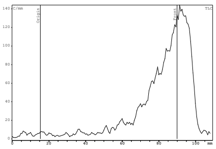	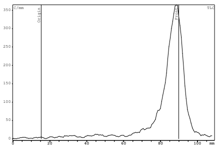	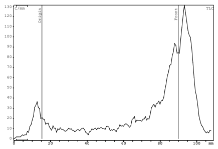	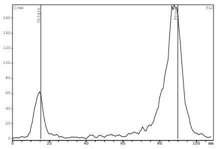
**[^{89}Zr]Zr-citrate in 1.0 M citric acid**	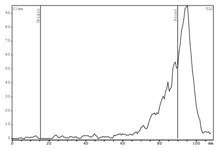	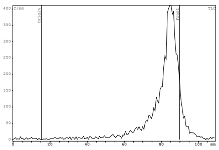	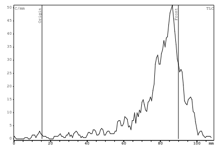	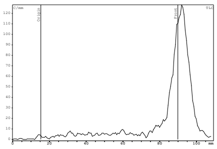

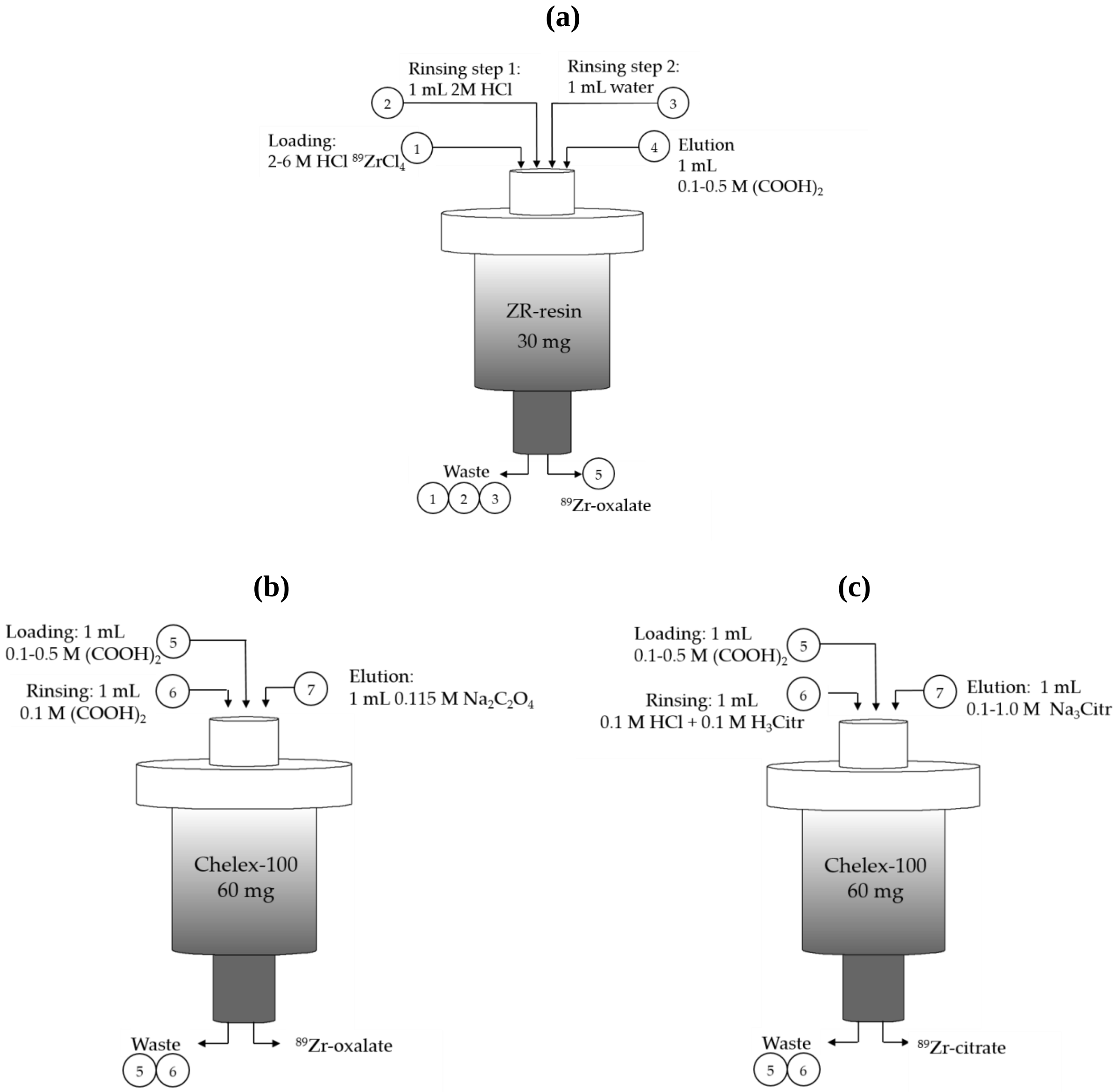

Figure S5. The scheme of methods proposed for preparation of zirconium-89 solutions for radiopharmaceutical purposes: **(a)** using ZR-resin only (zirconium-89 is obtained in 0.1-0.115 M oxalic acid); **(b)** using combination of ZR-resin and Chelex-100 (zirconium-89 is obtained in 0.1-0.116 M sodium oxalate); **(c)** using combination of ZR-resin and Chelex-100 (zirconium-89 is obtained in 0.1-1.0 M sodium citrate).

Table S6. Radio-TLC analysis results of [^{89}Zr]Zr-oxalate preparations during their storage.

	[^{89}Zr]Zr-oxalate in 0.1 M oxalic acid pH 6			
	**Only ZR-resin**		**ZR-resin + Chelex-100 resin**	
	Method 1	*Method 2*	*Method 1*	*Method 2*
Day 1	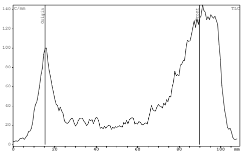	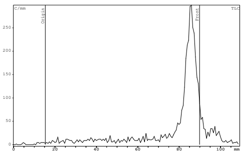	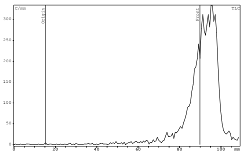	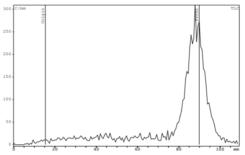
Day 5	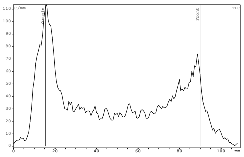	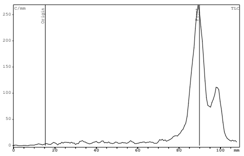	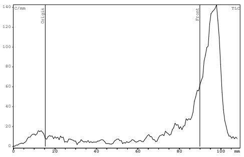	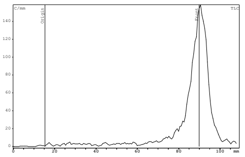
Day 10	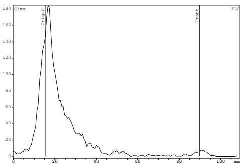	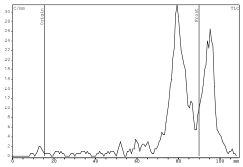	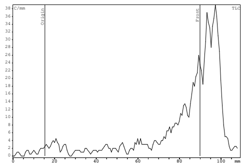	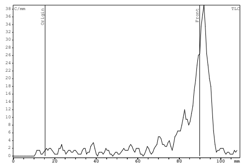

Table S7. Examples of analysis of the products of zirconium-89 reactions with DFO.

**[89Zr]zirconium formulation**	**Reaction mixture**	**Radio-chromatograms**		
		**iTLC-SG / 50 mM DTPA (pH 7)** *method 1*	**iTLC-SA / 50 mM EDTA (pH 5)**	**iTLC-SG / CH_3OH-H_2O (1:1), 4% TFA (v/v)** *method 2*
**[89Zr]Zr-oxalate (0.1 M oxalate anion) pH 6**	*pure*	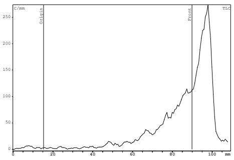	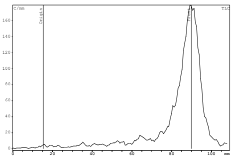	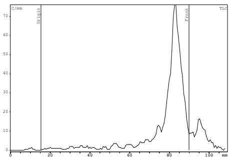
	+ 5.0 µg DFO	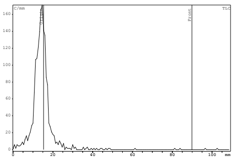	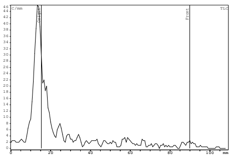	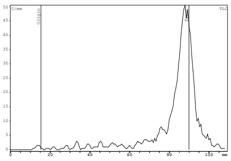
	+ 0.25 µg DFO	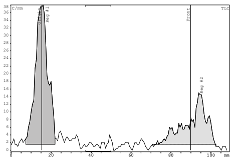	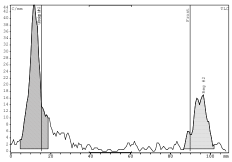	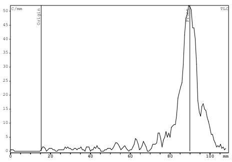
**[89Zr]Zr-citrate (0.1 M citrate anion) pH 6**	*pure*	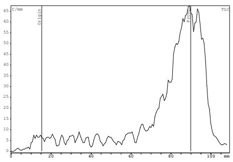	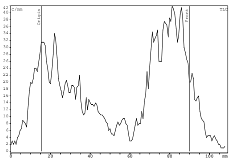	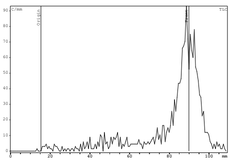
	+ 5.0 µg DFO	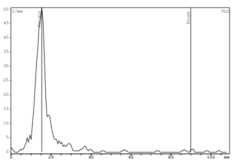	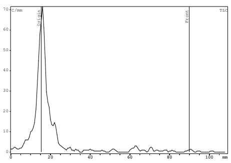	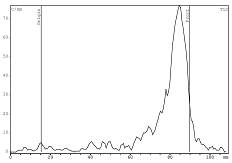
	+ 0.1 µg DFO	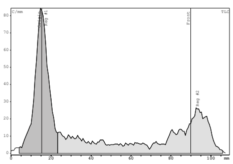	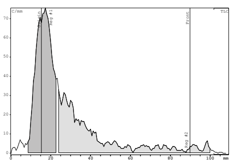	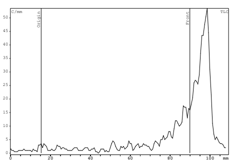

Table S8. The results of the analysis of various preparations of zirconium-89 while keeping the samples at room temperature and when heated.

**[89Zr]zirconium formulation**	**Temperature conditions**	**Radio-chromatograms**	
		**iTLC-SG / 50 mM DTPA (pH 7)** ***method 1***	**iTLC-SG / CH_3OH-H_2O (1:1), 4% TFA (v/v)** ***method 2***
**[89Zr]Zr-oxalate (0.1 M oxalate acid) pH 5.5**	*room temperature*	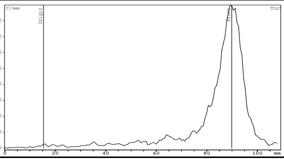	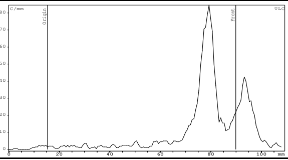
	95°C, 30 min	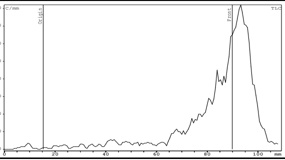	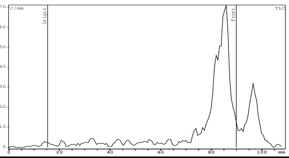
**[89Zr]Zr-citrate (0.1 M sodium citrate) pH 5.5**	*room temperature*	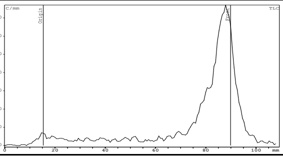	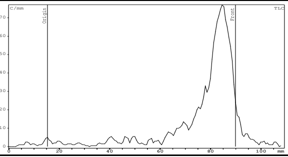
	95°C, 30 min	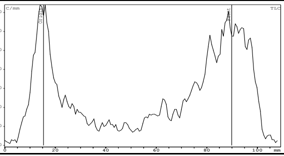	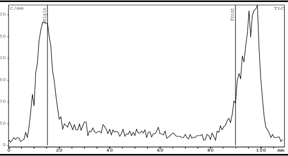
**[89Zr]Zr-citrate (1.0 M sodium citrate) pH 5.5**	*room temperature*	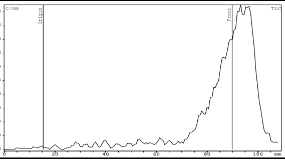	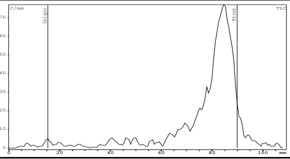
	95°C, 30 min	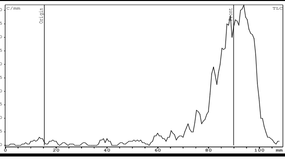	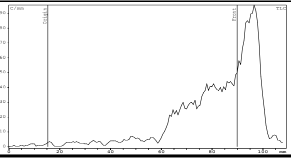

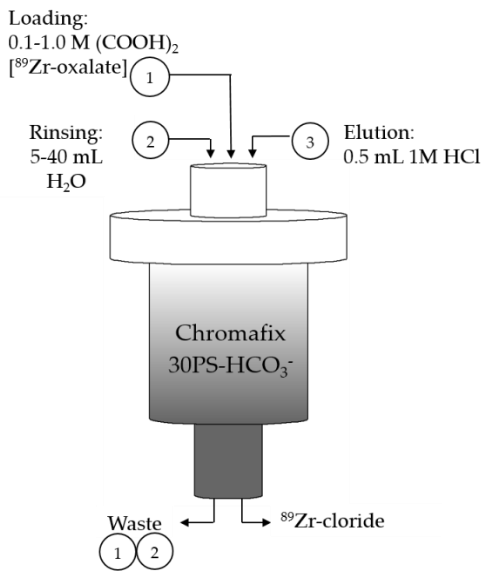

Figure S6. The scheme of zirconium-89 reconstitution from oxalic acid solution into hydrochloric acid solution using Chromafix 30PS-HCO_3 anion-exchange cartridge.

Table S9. Radio-TLC results (method 2) of zirconium-89 samples obtained in 1.0 M HCl using oxalate-free technology and using the reconstitution method from oxalic acid solution.

**[^{89}Zr]$ZrCl_4$ in 1 M HCl§**	**[^{89}Zr]Zr in 1.0 M HCl (neutralized to pH 6)§**	**[^{89}Zr]Zr in 1.0 M HCl after reconstitution**	**[^{89}Zr]Zr in 1.0 M HCl after reconstitution (pH 6, HEPES)**
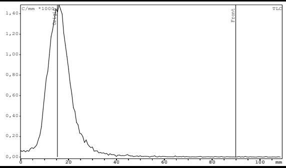	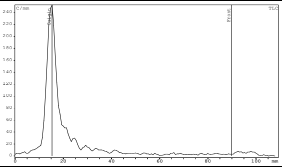	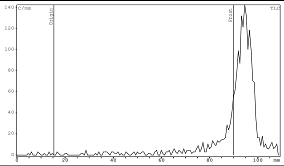	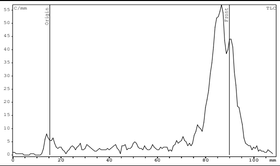

§ These samples were prepared from [^{89}Zr]$ZrCl_4$ in 5 M HCl initial solution obtained by the manufacturer using oxalate-free method (with Dowex 1×8 anion-exchange resin);.

Table S10. Radio-chromatograms in various systems. Zirconium-89 samples obtained in 1.0 M HCl using oxalate-free technology and those reconstituted from oxalic acid solution (pH = 6).

	Radio-TLC systems			
Sample characteristics	iTLC-SG / CH_3OH-H_2O (1:1), 4% TFA (v/v)	iTLC-SG / 50 mM DTPA (pH 7)	iTLC-SA / 50 mM EDTA (pH 5)	iTLC-SA / 0.5 M citric acid
[^{89}Zr]Zr in 1.0 M HCl (oxalate-free, neutralized to pH 6)	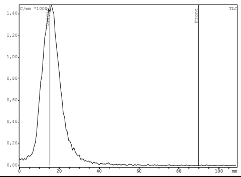	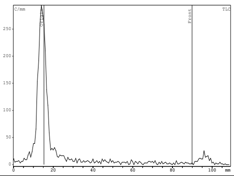	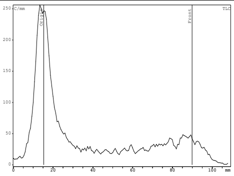	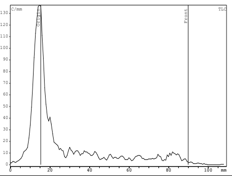
[^{89}Zr]Zr in 1.0 M HCl after reconstitution (pH 6, HEPES)	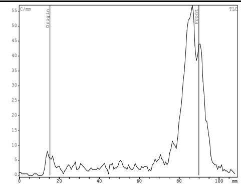	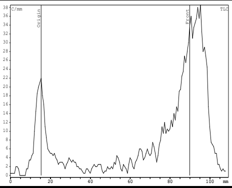	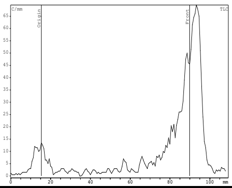	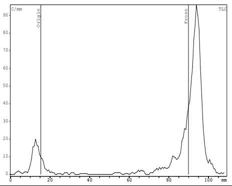
